# Pangenome analysis of *Proteus mirabilis* reveals lineage-specific antimicrobial resistance profiles and discordant genotype-phenotype correlations

**DOI:** 10.1128/aac.01768-25

**Published:** 2026-05-29

**Authors:** Namrata Deka, Aimee L. Brauer, Katherine Connerton, Blake M. Hanson, Jennifer N. Walker, Chelsie E. Armbruster

**Affiliations:** 1 Department of Microbiology and Immunology, Jacobs School of Medicine and Biomedical Sciences 12291 , Buffalo, New York, USA; 2 School of Nursing, Niagara University 5845, Buffalo, New York, USA; 3 Department of Epidemiology, UTHealth Houston School of Public Health 823773 , Houston, Texas, USA; 4 Center for Infectious Diseases, UTHealth Houston School of Public Health 823773 , Houston, Texas, USA; 5 Department of Microbiology and Molecular Genetics, McGovern Medical School, The University of Texas Health Science Center at Houston 12340, Houston, Texas, USA; Shionogi Inc., Florham Park, New Jersey, USA

**Keywords:** *Proteus mirabilis*, antimicrobial resistance, pangenome, antimicrobial susceptibility testing, multi-locus sequence typing, trimethoprim, sulfamethoxazole, chloramphenicol, urinary tract infection

## Abstract

Urinary tract infections (UTIs) impose a large healthcare burden, with escalating antimicrobial resistance (AMR), and treatment failure. *Proteus mirabilis* is an undercharacterized and challenging UTI pathogen due to intrinsic resistance and biofilm formation. To understand *P. mirabilis* population genomics, we combined pangenome analysis, *in silico* AMR prediction, and phenotypic antimicrobial susceptibility testing (AST) across 1,027 *P. mirabilis* genomes derived from human urine specimens. This revealed a mosaic pangenome driven by extensive accessory genome plasticity. Multilocus sequence typing (MLST) identified 213 MLSTs, with only 7% having ≥10 genomes, highlighting strain diversity. AMR gene profiles were largely lineage-specific, with 25% of genomes harboring resistances for >6 antimicrobial subclasses. ST135 was identified as a highly MDR lineage, with 95% of genomes carrying ≥16 resistance genes. Mobile genetic element (MGE) analysis of 22 clinical isolates with complete, reference-level genomes revealed that Tn7 transposons, IS26-mediated genomic islands, and class 1 integrons act as vehicles for high AMR gene dissemination, including IS26-mediated gene stacking within a *P. mirabilis* Genomic Resistance Island 1 (*PmGRI1*) in ST135 isolates. While the presence of genes like *aph(3′)-la* reliably predicted kanamycin resistance, discordance for antibiotics such as trimethoprim-sulfamethoxazole and chloramphenicol revealed that AMR gene stacking, regulatory context, and intrinsic mechanisms, like efflux pumps, modulate phenotypic outcomes. In summary, our study provides a comprehensive and phenotypic resolution of *P. mirabilis* AMR, establishing that resistance architecture is lineage-structured, MGE-driven, and phenotypically non-deterministic. We emphasize the need to shift toward a standardized, genome-informed surveillance framework to translate into diagnostic and therapeutic strategies.

## INTRODUCTION

Urinary tract infections (UTIs) are among the most common bacterial infections, with an estimated healthcare cost of >$3 billion in the United States (1–4). They are a leading cause of antimicrobial prescriptions in both outpatient and inpatient settings, with 50%–60% of women experiencing at least one UTI in their lifetime (5). UTIs can be classified as complicated UTI (cUTI) or uncomplicated UTI (uUTI) depending on the presence and absence of structural or functional abnormalities of the urinary tract, catheterization, or immunocompromised states (5). Recent epidemiological data estimate approximately 174,000 cases of uUTI and 2.38 million cases of cUTI annually (6, 7). Despite standard antibiotic therapy, treatment failure rates for UTIs remain prevalent, occurring in 16.7% of cases in adult female outpatients with cUTI, 27% of elderly men, and 15% of elderly women, often leading to recurrence, secondary infection, progression to pyelonephritis, or urosepsis (8, 9).

Among uropathogens, *Proteus mirabilis* is an important gram-negative bacterium and a major cause of UTIs, contributing to approximately 4.2% of uUTIs and 4.7% of cUTIs (5). Notably, *P. mirabilis* accounts for up to 45% of catheter-associated UTIs (CAUTIs) and 13%–21% of secondary bacteremia, making it a key pathogen in medical device-associated infections (5, 10, 11). *P. mirabilis* has a strong propensity for biofilm formation and causes encrustation of the catheter by deposition of struvite and apatite crystals, leading to persistent infections and catheter blockage (10, 12–15). These urinary stones can remain in the bladder as crystalline deposits despite multiple doses of antibiotic treatment, catheter changes, and even periods without catheterization (15–17). Infection with *P. mirabilis* can become fatal when it progresses to symptomatic CAUTI, acute bacteremia, and urosepsis due to its ability to disseminate from the bladder to other organs (14, 18).

Antimicrobial resistance (AMR) in UTI pathogens is an escalating global concern, with increasing reports of multidrug-resistant (MDR) strains complicating treatment (19). Treatment failure for *P. mirabilis*-associated UTIs has been linked to MDR, leading to prolonged hospitalization, increased healthcare costs, and higher morbidity (15, 19, 20). While antibiotic therapy remains the primary treatment approach, its efficacy is increasingly compromised by both intrinsic and acquired resistance mechanisms that limit therapeutic options (14, 21–23). The resistance to first-line UTI antibiotics such as trimethoprim-sulfamethoxazole (TMP-SMX), fluoroquinolones, and β-lactams is increasing, and the prevalence of ESBL genes like *blaTEM* and *blaCTX-M* further exacerbates resistance. Among the key drivers of AMR dissemination in *P. mirabilis* are mobile genetic elements (MGEs) such as SXT/R391 integrative and conjugative elements (ICEs) (19, 24, 25). However, characterization of MGEs in *P. mirabilis* UTI clinical isolates remains limited. Understanding the MGE landscape is essential for deciphering the mechanisms underlying horizontal gene transfer, the evolution and spread of MDR, and the discordance between genotypic predictions and phenotypic resistance.

Numerous studies have characterized the model *P. mirabilis* strain HI4320, originally isolated from the female urinary tract, and its pathogenic potential (10, 26). However, a major gap remains in our understanding of the diversity of strains isolated from recent clinical urine samples, either during infection or in the presence of a catheter. Multilocus sequence typing (MLST) provides an unambiguous method for characterizing bacterial isolates based on the sequences of internal fragments of typically 6–7 housekeeping genes. While previous studies in other bacterial species suggest that certain lineages correlate with varying levels of resistance and biofilm production, such associations in *P. mirabilis* remain largely unexplored. For example, in methicillin-resistant *Staphylococcus aureus* (MRSA), expression of the *ica* genes—critical for biofilm formation—varies among different STs, with certain STs showing higher *ica* gene expression, particularly in strains isolated from wound and catheter samples (27). If similar correlations exist in *P. mirabilis*, routine sequence typing of clinical isolates could serve as a valuable tool to predict AMR potential and pathogenicity.

Several typing schemes have been developed to characterize *P. mirabilis* strains, but no standard scheme is followed for clinical classification. Core genome MLST (cgMLST), a high-resolution genomic method, has identified 205 clonal groups (CGs) linked to severe UTIs and AMR (28). Historically, *P. mirabilis* strain differentiation has relied on methods such as bacteriophage typing, serotyping, and Dienes typing, but genomic approaches now offer superior discriminatory power (29–33). Even with the established role of *P. mirabilis* in CAUTI, the publicly available genome database is limited compared to other UTI pathogens (32, 34, 35). Furthermore, there remains a lack of integrated data linking sequence types (ST) to AMR gene carriage in urinary isolates using a standard classification framework. To address this gap, we focused specifically on genomes isolated from human urine, enabling a targeted analysis of AMR profiles and providing insights that may inform risk stratification for treatment failure and severe disease.

There are numerous ways to predict AMR, including the standardized clinical laboratory practices of testing resistance phenotypes to specific antibiotics, as well as genomic or machine learning methods to predict resistant gene expression (36). Genomic prediction tools can be useful for quick detection of AMR in clinical practice, and there may be a correlation between AMR gene prediction and phenotypic expression, but the nature of the correlation can vary depending on specific genes and the bacterial species. For instance, *Escherichia coli* frequently harbors resistance genes for β-lactams, sulfonamides, trimethoprim, and chloramphenicol on mobile genetic elements, leading to a stronger correlation between genotype and phenotype (37–39). In contrast, according to some studies, *P. mirabilis* carrying β-lactam and sulfonamide (*sul1* and *sul2*) genes exhibits this correlation less consistently, showing weaker alignment between genomic predictions and observed resistance profiles (37, 40–42).

Tools like AMRFinder and CARD are accurate *in silico* prediction tools; AMRFinder was able to validate the presence of genes pertaining to AMR phenotypes for *Salmonella enterica, Campylobacter spp*., and *E. coli* with 98.4% consistent predictions (43). Furthermore, a 99% concordance was found between phenotypic antimicrobial susceptibility testing and *in silico* prediction of AMR genes using whole-genome sequencing (WGS) in 1,321 isolates of *Salmonella enterica* from human infections in Canada (20). While WGS-based methods provide a promising avenue to predict AMR in other species, no equivalent variation exists for *P. mirabilis*. Hence, integrating genotypic AMR prediction and phenotypic MIC testing in diverse strains may offer a more comprehensive approach to predicting antimicrobial resistance in this species.

In this study, we conducted a comparative genomic analysis of *P. mirabilis* using 1,001 publicly available human urine isolate sequences to characterize the pangenome, AMR gene profiling, and *in silico* MLST typing. We benchmarked this against 26 clinical isolates collected from patients with long-term indwelling catheters (15, 17). Our analysis revealed that AMR gene profiles can link to specific lineages rather than isolation source, with isolates of the same MLST clustering together and exhibiting related resistant genes. We found that the ST135 lineage is a hotspot for resistance gene acquisition in our data set, driven by the integration of MGEs such as IS26 and the *PmGRI1* genomic island with multiple overlapping resistance genes. We further identified a high rate of discordance between genotype-based susceptibility prediction and phenotypic resistance. This discrepancy can be partially attributed to uncharacterized MGEs and efflux pump mechanisms, highlighting the limitations of relying on gene detection only for resistance profiling. Our findings underscore the need for integrating lineage-specific MGE surveillance with phenotyping testing to guide treatment options and reduce the overuse of broad-spectrum antibiotics in managing *P. mirabilis* CAUTI infections.

## MATERIALS AND METHODS

### Patient population and sample collection

To investigate whether *P. mirabilis* MLSTs correlate with AMR patterns, we assessed *P. mirabilis* genomes from three separate cohorts: publicly available genomes from NCBI (Table S1), nursing home residents with long-term catheters (CEA isolates), and community-dwelling catheterized patients (HUC isolates).

For comparison of *P. mirabilis* genomes, we sought to query all publicly available high-quality genomes of *P. mirabilis* isolates obtained from urine samples in hospital environments in the United States. Using advanced search parameters, publicly available raw genome data were retrieved from the NCBI database. The initial search, conducted in October 2024, yielded 1,337 hits using the terms “*Proteus mirabilis*,” isolation source “urine,” and host “*Homo sapiens*.” Filtering the results to include only isolates originating from the “United States” reduced the data set to 1,152 genomes. The data set was further refined by only including whole-genome sequencing data. These filters resulted in a final data set of 1,064 (Table S1) genomes, which were subsequently downloaded from NCBI. Following FastQC v0.11.9-Java-11 analysis and adapter trimming using trimmomatic v0.39-Java-11.0.16 (44, 45); 1,026 genomes were retained for downstream analyses. Samples were excluded if they exhibited poor quality, had high GC content, high read duplication rates, or if paired-end read files were missing (46 samples). Raw paired-end read sequences were assembled using SPAdes v3.15.5 (46). After genome assembly, we assessed quality and completeness using QUAST (Table S2), selecting 1,001 high-quality genomes with >95% completeness and fewer than 1,000 contigs that were annotated with Prokka v1.14.5 to facilitate downstream analyses (47, 48).

Isolates designated 101–210 were cultured from urine specimens collected during a prospective cohort study on asymptomatic catheter-associated bacteriuria at two nursing homes in Buffalo, New York, USA, between July 2019 and March 2020 (15). Inclusion criteria required participants to be 21 years or older at the time of consent and have an indwelling urinary catheter in place for at least 12 months at the start of the study. Study participants underwent an initial enrollment visit, followed by weekly study visits for up to 7 months. Most of the study participants were White (79%) and male (79%), had an average age of 65 years, and 68% had suprapubic catheters (15). Participants exhibited a high degree of functional dependence in activities of daily living, with the most common comorbidities including neurogenic bladder (74%), hemiplegia (42%), diabetes (32%), renal disease (32%), multiple sclerosis (26%), and chronic heart failure (26%) (15). Urine specimens were cultured on MacConkey agar and Columbia Nutrient Agar, and gram-negative bacteria were identified to the species level using API-20E test strips (BioMérieux). *P. mirabilis* species designation was further confirmed based on characteristic swarming motility on blood agar plates (Hardy Diagnostics) and whole genome sequencing (Table S3).

Additional *P. mirabilis* isolates with designations of “HUC” were obtained from the lab of Scott J. Hultgren, Washington University School of Medicine (17). These isolates were derived from urine samples collected from community-dwelling catheterized patients during routine catheter removals in the urology clinic as part of their standard care. Most of the participants were male (64%), with an average age of 64 years. Inclusion criteria required participants to be 18 years or older at the time of consent and scheduled for the removal of an indwelling urinary device, including urethral and suprapubic catheters or ureteral stents. The primary indications for catheterization included bladder outlet obstruction (33%), peripheral or central neuropathy (40%), rectovesical or rectourethral fistula (7%), stress urinary incontinence (7%), atonic bladder (11%), and temporary post-operative complications (2%) (17). Bacterial colonies were distinguished based on morphology, size, and color, and 1–9 single colonies per sample were selected for DNA extraction. *P. mirabilis* isolates were identified via 16S rRNA sequencing and confirmed by whole genome sequencing (Table S3).

### Whole genome sequencing of clinical isolates

Freezer stocks were streak-plated on MacConkey agar to isolate single colonies of *P. mirabilis*; 5 mL of Low Salt Luria-Bertani (LSLB; 0.1 g/L NaCl, 10 g/L Tryptone, 5 g/L yeast extract) broth was inoculated with a single colony and incubated for 16 h at 37°C with shaking. Genomic DNA was extracted using the DNeasy Blood and Tissue Kit (Qiagen, Germantown, Maryland, USA) according to the manufacturer’s instructions with specific modifications to ensure high purity of DNA; samples were incubated in proteinase K for 2 h instead of 10 min, and three washes of AW2 buffer were conducted to ensure high-quality DNA free of salts. Samples were sent to SeqCoast Genomics for whole genome sequencing (WGS). All samples were prepared using the Illumina DNA Prep tagmentation kit and IDT for Illumina Unique Dual Indexes. Sequencing was performed on the Illumina NextSeq2000 platform using a 300-cycle flow cell kit to produce 2 × 150 bp paired reads, as previously described (49); 1%–2% PhiX control was spiked into the run to support optimal base calling. The sequencing data have been uploaded to BioProject PRJNA1367153: *P. mirabilis* genome sequences from human urine sources in the United States. Read trimming and run analytics were performed using trimmomatic v0.39.0 (45). For long read sequencing, Oxford Nanopore Technologies Native Barcoding Kit (#SQK-NBD114) and long fragment buffer were used to promote longer read lengths of DNA. DNA was sequenced on the PromethION 2 Solo platform using a FLO-PRO114M Flow Cell (vR10.4.1). Sequencing was performed with a translocation speed of 400 bps. Base calling was done on the GridION using the super-accurate basecalling model with barcode trimming enabled.

Assembly of short reads from the clinical isolates and the genomes acquired from the NCBI database was performed using spades v3.15.5, while hybrid assembly of long and short reads from clinical isolates was performed using Unicycler v0.5.0 (46). Here, long reads were used to assemble the genome structure, and the short reads were used to polish the genome. The assemblies were quality-checked using QUAST v5.2.0, selecting genomes with <500 contigs for only short-read assembled genomes and <3 contigs for hybrid (Table S3). If sequences were not of good quality, they were further polished using pilon/1.23-Java-11.0.16 (50). Genomes that passed quality filtering were annotated using Prokka v1.14.5 (48).

### Multi-locus sequence typing (MLST) and phylogenetic trees

Multilocus sequence typing (MLST) analysis was conducted to identify the sequence types (STs) present in the data set using MLST software, which incorporates the PubMLST database (51, 52). The annotated genomes were then fed into roary v3.13.0 using a core threshold cluster of 99% to identify their pangenome profile and develop a gene presence/absence matrix (53). The filtered core genome alignment was constructed using FastTree v2.1.11. The resulting newick file was visualized as a mid-rooted phylogenetic tree using the iTOL website and annotated with source and antibiotic metadata files (54, 55).

### Drug resistance gene prediction

AMRFinderPlus (using the Bacterial Antimicrobial Resistance Reference Gene Database) and CARD (The Comprehensive Antibiotic Resistance Database) were used to predict AMR genes from the coding sequence of the *P. mirabilis* genomes, including the 26 CEA and HUC strains and clinical reference strain HI4320 (36, 43).

Mobile genetic elements were characterized in the hybrid-sequenced clinical isolates using multiple approaches. Insertion sequence (IS) elements were identified using ISEScan v1.7.3 (56). Prophage regions were predicted using PHASTER (PHAge Search Tool Enhanced Release) (57–59) and classified as intact (score >90), questionable (70–90), or incomplete (<70). Plasmid replicons were detected using ABRicate v1.0.1 with the PlasmidFinder database (≥80% identity, ≥60% coverage) (60, 61). Integrative and conjugative elements (ICEs) were identified via ICEberg 3.0 (62). Spearman’s rank correlation in R v4.3.0 assessed associations between IS abundance and AMR gene content and virulence factors, with significance set at *P* < 0.05.

### Antibiotic susceptibility testing (AST)

The clinical strains were grown overnight in 5 mL LSLB broth at 37°C with shaking. Antibiotic solutions, such as tetracycline HCl (Research Products International [RPI], CAS: 64-75-5), chloramphenicol (RPI, CAS: 56-75-7), streptomycin (RPI, CAS: 3810-74-0), streptothricin (Goldbio, CAS: 96736-11-7), sulfonamide (sulfamethoxazole, Cayman Chemical Company, CAS: 723-46-6), trimethoprim (Cayman Chemical Company, CAS: 738-70-5), ampicillin (RPI, CAS: 69-53-4), and kanamycin (RPI, CAS: 8063-07-8), were prepared in Mueller-Hinton Broth (MHB) at a concentration range encompassing the sensitive-to-resistant spectrum according to Clinical Laboratory Standards Institute (CLSI) guidelines for *Enterobacteriales* (63). Stock solutions of all the antibiotics were prepared and stored at −20°C, and working dilutions were made fresh from the stock immediately prior to testing.

One milliliter of the overnight cultures was centrifuged at 1,000 × *g* to pellet the cells. The supernatant was removed, and the pellets were resuspended in 0.9% saline to wash away residual LB. The inoculum was adjusted to 4 × 10^5^ CFU/mL in MHB. One antibiotic with increasing concentrations was tested per 96-well plate, with four technical and duplicate biological replicates. The plates were incubated at 37°C with double-orbital shaking in Biotech Synergy H1, and the OD_600_ was measured every 15 min for 20 h to monitor bacterial growth with an OD_600_ threshold set at 0.15.

### Statistical analysis

Logistic regression models were used to explore AMR gene carriage as a function of sequence type, geographic location of isolation, and year of isolation. Data were analyzed using Stata/IC version 15.1 (StataCorp LP, College Station, TX).

## RESULTS

To assess the population structure and genetic diversity of *P. mirabilis* in the urinary tract, we analyzed 1,001 publicly available genomes isolated from human urine specimens (Table S1). The genomes downloaded from the NCBI were predominantly derived from clinical settings, focusing on medical surveillance and characterization of urinary samples.

Key contributing sources include research from Washington University School of Medicine (WUSM), the University of Pittsburgh Enhanced Detection System for Healthcare-Associated Transmission (EDS-HAT) study, and national antimicrobial surveillance programs such as the Centers for Disease Control’s Healthcare-Associated Infection Sequencing (CDC HAI-Seq) Gram-Negative Bacteria Study and studies on the human urinary microbiome. Among the genomes, contig counts ranged from 60 to 973, with an average of 258 and a median of 180 contigs. The GC content averaged 38.76%, with a median of 38.69%, making them suitable for downstream analyses (Table 1; Table S2). While all sequences were confirmed to be derived from urine samples, the clinical context of these specimens—whether they were associated with asymptomatic bacteriuria or infection—is not known.

**TABLE 1:** Summary statistics of *P. mirabilis* genome sequences

Metric	Average	Max	Min	Median
Publicly available *P. mirabilis* genomes isolated from urine				
# Contigs	258.43	973	60	180
Total length	4,020,761.51	8,382,181	3,667,799	3,992,140
N50	174,294.28	1,994,055	4,981	174,571
GC content	38.76	43.24	37.57	38.69
Clinical *P. mirabilis* genomes isolated from catheterized individuals				
# Contigs	66.52	98	2	69
Total length	4,015,048.48	4,165,371	3,795,264	4,015,495
N50	317,358.93	4,063,606	119,871	182,416
GC content	38.74	38.98	38.38	38.78

**Fig 1:**
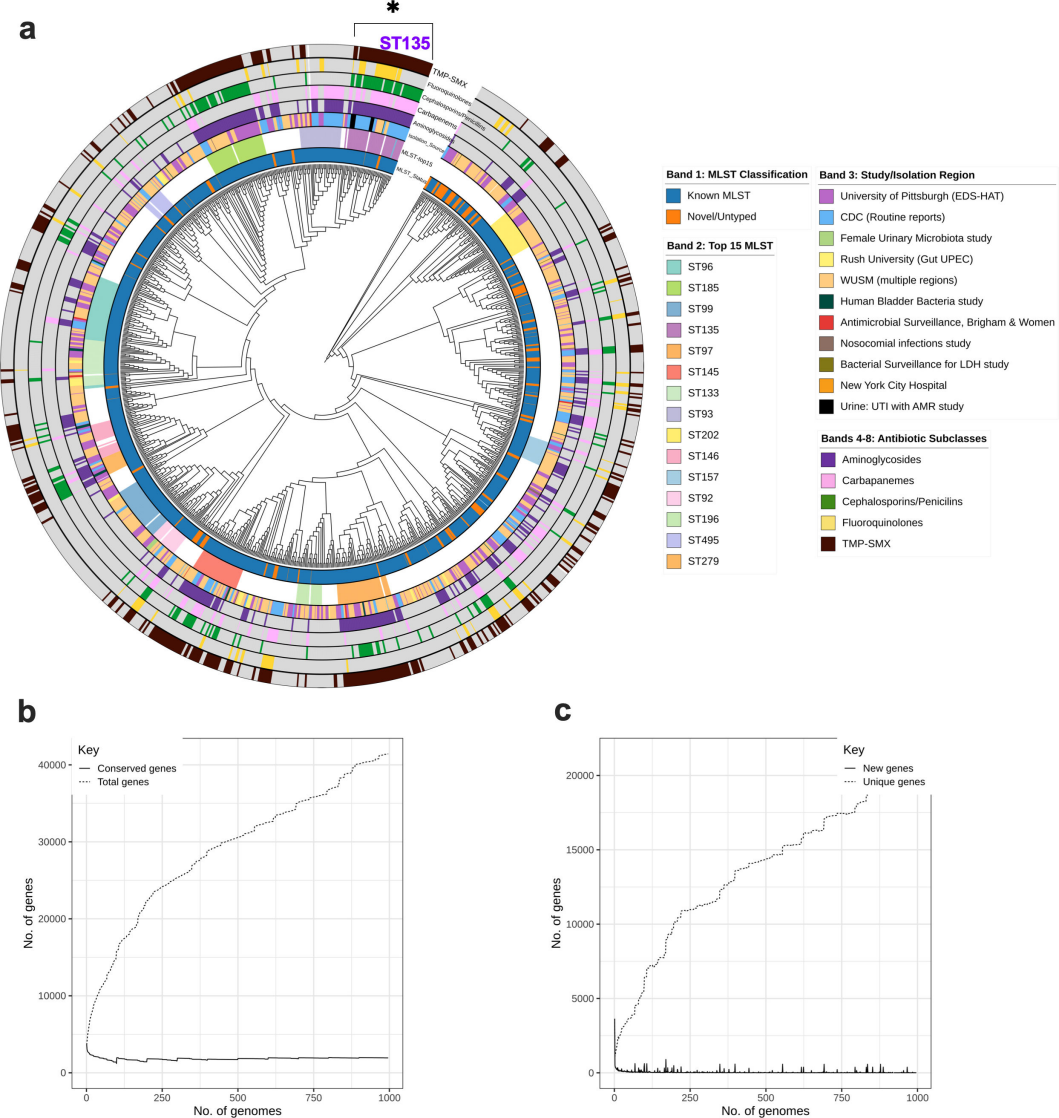
Phylogenetic tree and pangenome analysis of publicly available *P. mirabilis* genomes from human urine samples. (**a**) Maximum-likelihood phylogenetic tree of 1,001 *P. mirabilis* human urine genomes with metadata arranged as concentric bands. The first two bands display multi-locus sequence type (ST), and the third band indicates the study source in the NCBI database associated with the Bioproject. Bands 4–8 indicate the presence (colored bars) or absence (gray) of resistance genes across different antibiotic classes. Distinct clusters were observed that either had resistance genes for nearly all classes of antibiotics or lacked them entirely, which was dependent on ST and independent of geographical source. (**b**) Roary collector curve showing conserved genes (~1,700 core genome) present in all of the genomes analyzed as well as the number of total genes, which increases as new genomes are added, indicating an open pangenome. (**c**) Roary collector curve showing unique vs. new gene accumulation across all *P. mirabilis* genomes. Here, the total unique genes are > 15,000, indicating massive accessory gene diversity and an open pangenome. Early genomes introduce a lot of new genes before dropping out. However, after the addition of > 250 genomes, new genes (vertical lines) are still accumulated, indicating horizontal gene transfer (HGT), mobile genetic elements, or prophage insertions.

Phylogenetic analyses (mid-rooted tree) revealed a monophyletic, star-like phylogeny, with a central ancestral node from which several peripheral branches radiate (Fig. 1a), which may indicate recent diversification events. When considering branching, a subset of genomes branched out, creating three clades: *clade 1* (964 genomes, dominant), *clade 2* (5 genomes), and *clade 3* (32 genomes) (Fig. S1). This breakdown is consistent with previously reported intra-species core genome variation in *P. mirabilis* (32, 33, 35). For example, Potter et al. studied a cohort of 2,060 *P. mirabilis* genomes collected worldwide from multiple anatomical sources and observed that the essential core genes remained relatively conserved with three divergent sub-species (32).

### Pan-genomic analysis of *P. mirabilis* reveals a mosaic structure

To characterize the pangenome of *P. mirabilis* from human urine samples, we assessed its structural composition, including core, shell, and cloud gene categories. In the 1,001 genomes analyzed, a total of 41,121 gene families were identified. Of these, the core genome (genes conserved in >99% of the samples analyzed) is small with 1,937 genes, indicating a high degree of conservation. Considering that a typical *P. mirabilis* genome harbors roughly 4,600–4,900 coding sequences (CDS); the core genome accounts for approximately 40%–45% of the average gene content. This indicates a highly conserved genomic backbone, with 4.7% of it supporting essential cellular functions, coexisting with an expansive and dynamic accessory genome (Fig. S1). The shell of the 1,001 genomes contained 1,749 genes (4.08%), and 531 soft core genes were shared among a subset of strains that likely contribute to niche-specific adaptations. The majority of genes (37,204 genes, 90%) were classified as cloud genes, making the accessory component the largest in the pangenome. Having a small, stable core and a large, variable accessory genome likely represents strain-specific differences driven by horizontal gene transfer.

The pangenome exhibited an open conformation representing a mosaic pattern (Fig. S1), with the size expanding as additional genomes were analyzed (Fig. 1b). This expansion reflects the continuous acquisition of novel gene families, highlighting the dynamic nature of the *P. mirabilis* genome and its capacity for adaptation through horizontal gene transfer (Fig. 1c). The stability of core genes is crucial for the consistent expression of virulence factors, while the expanding accessory genes may help adapt to polymicrobial environments and/or antibiotic pressures. These findings indicate the genetic plasticity of *P. mirabilis* isolated from urine and provide a foundation for future studies on its pathogenic potential and AMR mechanisms in the urinary tract.

### Publicly available *P. mirabilis* genomes comprise 213 defined and 118 undefined MLSTs

The specific genes used in MLST of *P. mirabilis* are a combination of housekeeping genes *atpA, dnaJ, mdh, pyrC, recA,* and *rpoD* (51, 52). We identified a total of 213 defined STs and approximately 118 novel or undefined genomes, reflecting the diversity of *P. mirabilis* human urine isolates within the data set (Fig. 1). The untyped MLSTs have been submitted to the PubMLST database to update the schema in *P. mirabilis*.

**Fig 2:**
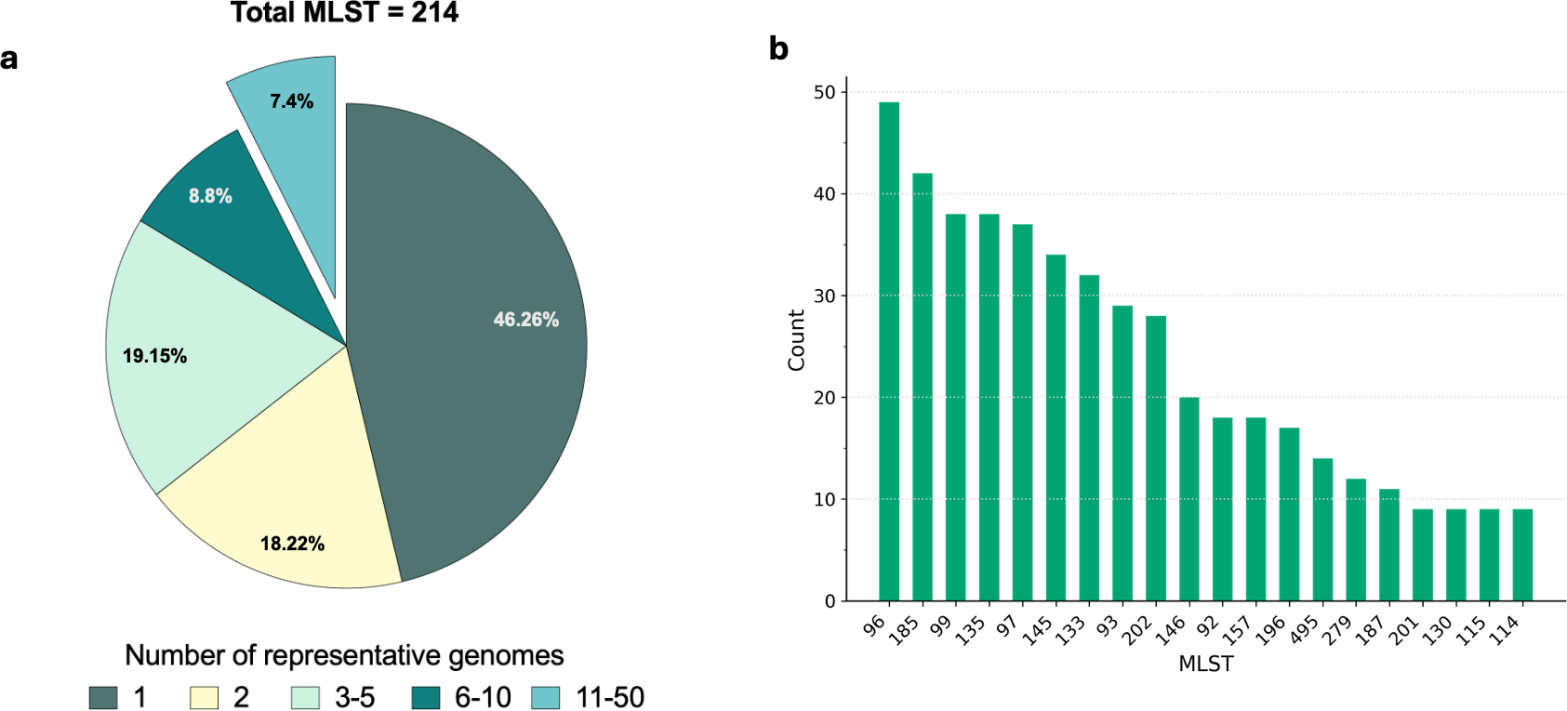
Sequence type distribution of publicly available *P. mirabilis* genomes from human urine samples. (**a**) Pie chart illustrating the distribution of MLSTs grouped by their sample count frequency. (**b**) Bar plot showing the top 20 MLSTs based on the sample count.

A detailed analysis of ST prevalence within the data set revealed that certain STs were more frequently observed. The distribution of MLSTs across the data set was highly skewed (Fig. 2a; Tables S4 and S5), with 46.26% of the STs having only one representative genome in the data set, while ~7% of the STs had ≥10 genomes (Fig. 2a). Many genomes in the same ST came from the same study/location, but it was not the rule as the top 15 overrepresented STs show mixed isolation sources (Fig. 1a). The uneven ST distribution is further visualized in a bar chart of the top 20 MLSTs, highlighting the dominance of a few clonal lineages across sampled populations (Fig. 2b). ST96 was the most abundant (49 genomes; 4.90%), followed by ST185 (42 genomes; 4.20%), ST99 (38 genomes; 3.80%), ST135 (38 genomes; 3.80%), and ST97 (37 genomes; 3.70%). As expected, genomes from each of these STs clustered together by lineages, even if isolated from different geographical locations and reported in different studies (Fig. 1a).

### Phylogenetic analysis reveals shared lineages and novel variants across clinical *P. mirabilis* isolates

**Fig 3:**
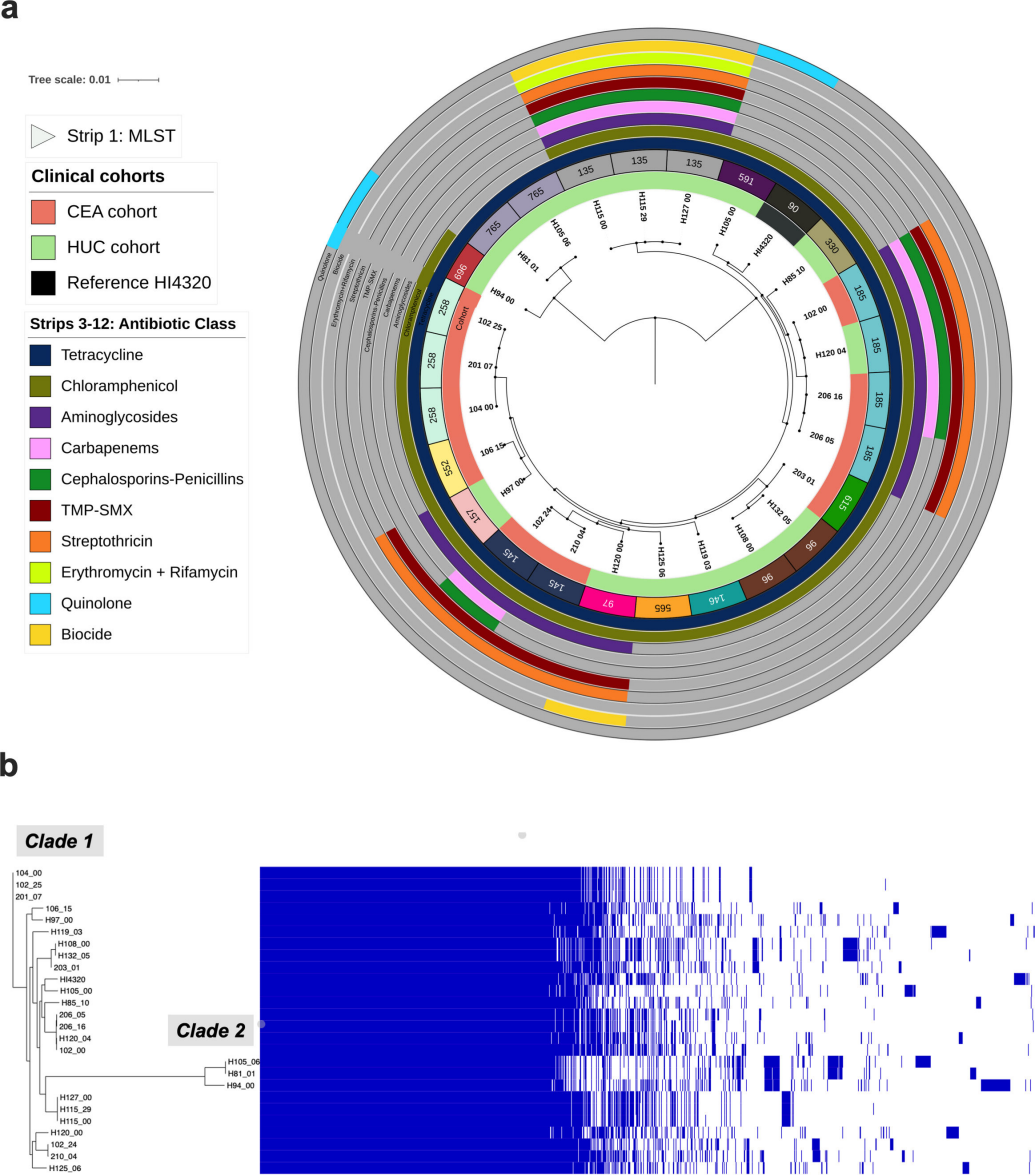
Phylogenetic tree and pangenome analysis of *P. mirabilis* strains isolated from individuals with indwelling urinary catheters. (**a**) A mid-rooted maximum likelihood phylogenetic tree of clinical *P. mirabilis* isolates collected from patients with long-term urinary catheters. The phylogenetic tree displays the participant ID, the cohort ID (strip1), and the multi-locus sequence type (ST, strip 2). Many of the isolates cluster by ST, but not by geographical location. (**b**). Mosaic pangenome with presence/absence matrix of core-genome phylogenetic tree for 26 *P. mirabilis* clinical isolates collected from patients with long-term urinary catheters.

To further examine the clinical relevance of *P. mirabilis* MLSTs, we next sequenced and analyzed a collection of 25 clinical isolates from two distinct cohorts (CEA isolates collected from the urine of nursing home residents with long-term indwelling catheters, and HUC isolates collected from the urine of catheterized community-dwelling individuals during routine catheter changes), as well as the reference strain HI4320 that was previously isolated from the urine of a catheterized nursing home resident (Fig. 3a). The analysis revealed a population structure forming two primary clades, termed *clade 1* (23 isolates) and *clade 2* (3 isolates) (Fig. 3b). Lack of the third clade identified in the 1,001 genomes was expected considering its low frequency (0.5%) and the small sample size of the clinical isolate collection.

Phylogenetic clustering was in accordance with the core genome and gene content irrespective of the clinical origin (HUC vs. CEA, Fig. 3a). Across the 26 isolates, we identified 15 distinct STs; 10 STs were identified in the HUC cohort, 4 in the CEA cohort, and 1 represented solely by HI4320. Six of the 10 overrepresented STs in the NCBI genome data set were also represented among the clinical isolates, namely STs 96, 97, 135, 145, 146, and 185. ST185 was observed in both the CEA and HUC cohorts (H120-04 and 102-00, 206-16), demonstrating genetic overlap across different geographic locations. The two most prevalent STs in the NCBI data set were also the most prevalent in the clinical isolates: ST185 (three isolates, 12%) and ST96 (three isolates, 12%). Their presence confirms that our clinical cohort captured high-impact, epidemiologically relevant STs rather than rare or sporadically enriched variants, even with a limited sample size. The full summary of isolated sources and STs is provided in Table 2 and Table S3.

**TABLE 2:** *P. mirabilis* clinical isolates used in this study

Isolate ID	Study	Species	MLST	Patient designation in cited publication	Isolation week
102-00	CEA	*P. mirabilis*	185	Participant A (15)	Visit 0
102-24	CEA	*P. mirabilis*	145	Participant A (15)	Visit 24
102-25	CEA	*P. mirabilis*	258	Participant A (15)	Visit 25
104-00	CEA	*P. mirabilis*	258	Participant C (15)	Visit 0
106-15	CEA	*P. mirabilis*	552	Participant H (15)	Visit 15
201-07	CEA	*P. mirabilis*	258	Participant G (15)	Visit 7
203-01	CEA	*P. mirabilis*	615	Participant F (15)	Visit 1
206-05	CEA	*P. mirabilis*	185	Participant D (15)	Visit 5
206-16	CEA	*P. mirabilis*	185	Participant D (15)	Visit 16
210-04	CEA	*P. mirabilis*	145	Participant J (15)	Visit 4
H81-01	HUC	*P. mirabilis*	765	Patient 81-urine (17)	Visit 1
H85-10	HUC	*P. mirabilis*	330	Patient 85-urine (17)	Visit 10
H94-00	HUC	*P. mirabilis*	696	Patient 94-urine (17)	Visit 0
H97-00	HUC	*P. mirabilis*	157	Patient 97-urine (17)	Visit 0
H97-11	HUC	*P. mirabilis*	157	Patient 97-urine (17)	Visit 11
H105-00	HUC	*P. mirabilis*	591	Patient 105-urine (17)	Visit 0
H105-06	HUC	*P. mirabilis*	765	Patient 105-urine (17)	Visit 6
H108-00	HUC	*P. mirabilis*	96	Patient 108-urine (17)	Visit 0
H115-00	HUC	*P. mirabilis*	135	Patient 115-urine (17)	Visit 0
H115-05	HUC	*P. mirabilis*	135	Patient 115-urine (17)	Visit 5
H119-03	HUC	*P. mirabilis*	146	Patient 119-urine (17)	Visit 3
H120-00	HUC	*P. mirabilis*	97	Patient 120-urine (17)	Visit 0
H120-04	HUC	*P. mirabilis*	185	Patient 120-urine (17)	Visit 4
H125-06	HUC	*P. mirabilis*	565	Patient 125-urine (17)	Visit 6
H127-00	HUC	*P. mirabilis*	96	Patient 127-urine (17)	Visit 0
H132-05	HUC	*P. mirabilis*	96	Patient 132-urine (17)	Visit 5
HI4320	Reference	*P. mirabilis*	90	Refseq: GCA_000069965.1_ASM6996v1_genomic	–^*a*^

^*a*^ –, not applicable.

### *P. mirabilis* urine isolates possess resistance factors to multiple subclasses of antibiotics

To understand how AMR complicates *P. mirabilis* UTI treatment and contributes to the global public health burden, we examined the AMR profiles of the 1,001 publicly available *P. mirabilis* genomes. A total of 24 antibiotic resistance subclasses were identified, reflecting the diverse mechanisms by which this pathogen can evade antibiotics. The most prevalent genes were those conferring resistance to tetracycline and chloramphenicol, observed in nearly all genomes (Table S6).

**Fig 4:**
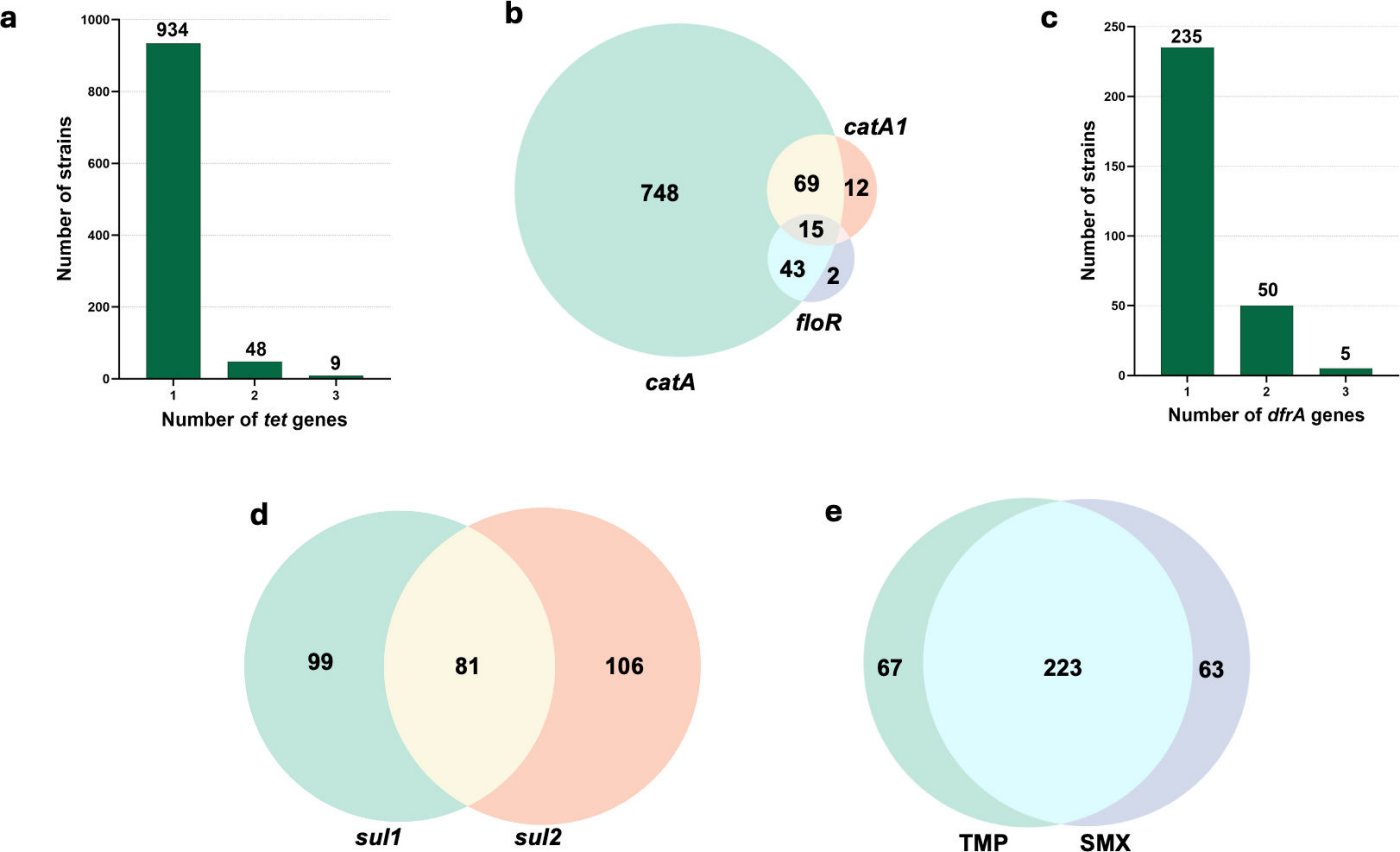
AMR gene carriage in publicly available *P. mirabilis* genomes. (**a**) Number of *P. mirabilis* strains in the NCBI data set carrying 1, 2, and 3 tetracycline resistance (*tet*) genes. (**b**) Three-way Venn diagram exhibiting the shared chloramphenicol resistance genes (*catA, catA1,* and *floR*) by all the *P. mirabilis* isolates in the NCBI data set. (**c**) Number of *P. mirabilis* strains in the NCBI data set carrying 1, 2, and 3 trimethoprim resistance (*dfrA*) genes. (**d**) Venn diagram of shared sulfonamide resistance genes (*sul1* and *sul2*). (**e**) Venn diagram of shared trimethoprim (TMP) and sulfonamide (SMX) resistance genes.

*P. mirabilis* is known to have intrinsic resistance to tetracycline; hence, it was not surprising that tetracycline resistance genes were detected in 991 (99.0%) of the 1,001 genomes, with *tetJ* specifically encoded by 985 of those 991 (99.5%) (Table 3). Several other tetracycline resistance determinants, including *tetA, tetB, tetC*, and *tetD*, were detected at lower frequencies. Interestingly, 934/991 (94.2%) of the genomes carried only a single *tet* gene, while 48 (4.8%) carried 2, and 9 (0.9%) carried 3 *tet* genes (Fig. 4a). Notably, a small number of genomes (11, 1.1%) had no detectable tetracycline resistance genes. This could represent a true absence of the genes, or it could be a result of fragmented assemblies or sequence dissimilarity not found in the AMR prediction database.

**TABLE 3:** AMR subclass count and percentage in *P. mirabilis* genomes^*a*^

Resistance subclass	Count (NCBI)	Genes detected (NCBI)	Percentage (1,001 NCBI)	Count (clinical)	Genes detected (clinical)	Percentage (26 clinical)	NCBI/Clinical Comparison
Amikacin/kanamycin / quinolone / tobramycin	11	*aac(6′)-Ib, aph(3′)-IIIa, aph(3′)-VI, aph(3′)-VIb, aph(3′)-XV*	7.19%	1	*aac(6′)-Ib-cr5*	3.70%	Lower in clinical
Beta-lactam	185	*blaCARB-2, blaOXA, blaOXA-1033, blaOXA-2, blaOXA-9, blaTEM, blaTEM-1, blaTEM-2, blaTEM-30, hugA*	18.46%	5	*blaTEM-1*	18.52%	Similar in prevalence
Cephalosporin	90	*ampC, blaCMY-2, blaCMY-6, blaCTX-M-14, blaCTX-M-15, blaCTX-M-2, blaCTX-M-3, blaCTX-M-55, blaCTX-M-65, blaCTX-M-91, blaDHA, blaDHA-1, blaFOX-5, blaOXA-1, blaOXA-10, blaPDC-3, blaPER, blaPER-1, blaSHV-12, blaSHV-31, blaTEM-10, blaTEM-155, blaVEB-6*	8.98%	1	*blaOXA-1*	3.70%	Lower in clinical
Chloramphenicol	891	*catA, catA1, catA2, catA3, catB11, catB2, catB3, catB7, catB8, cmlA, cmlA5, cmlA6*	88.92%	23	*catA, catA1, catB3, floR*	85.19%	Similar in prevalence
Erythromycin	36	*ere(A), ere(B) mph(E*)	3.50%	1	*ere(A*)	3.70%	Similar in prevalence
Kanamycin	137	*aph(3′)-IIb, aph(3')-Ia*	13.67%	7	*aph(3′)-Ia*	25.93%	Higher in clinical
Quaternary ammonium	192	*qacE, qacEdelta1*	19.16%	3		11.11%	Lower in clinical
Quinolone	68	*qnrA, qnrA1, qnrB19, qnrB2, qnrB4, qnrD, qnrD1, qnrD2, qnrS1, qnrVC1*	6.79%	2	*qnrD*	7.41%	Similar in prevalence
Rifamycin	28	*arr-2, arr-3*	2.79%	1	*arr-3*	3.70%	Similar low prevalence
Streptomycin	352	*aadA1, aadA10, aadA16, aadA2, aadA3, aadA31, aadA5, ant(6)-Ia, aph(3'')-Ib, aph(6)-Id*	35.13%	10	*aadA1, aph(6)-Id, aph(3'')-Ib, aadA5, aadA2*	37.04%	Similar in prevalence
Streptothricin	282	*sat2*	28.14%	10	*sat2*	37.04%	Higher in clinical
Sulfonamide	286	*sul1, sul2*	28.54%	6	*sul1, sul2*	22.22%	Slightly lower in clinical
Tetracycline	991	*tet(59), tet(A), tet(B), tet(C), tet(D), tet(G), tet(H), tet(J), tet(L), tet(M), tet(Y*)	98.90%	26	*tet(J), tet(A), tet(C*)	100%	Nearly universal presence in both
Trimethoprim	290	*dfrA1, dfrA10, dfrA12, dfrA14, dfrA16, dfrA17, dfrA19, dfrA27, dfrA32, dfrA42, dfrA46, dfrA5, dfrA7*	28.94%	10	*dfrA1, dfrA17, dfrA32*	37.04%	Higher in clinical
Azithromycin / clarithromycin / clindamycin / erythromycin / telithromycin	1	*erm(A*)	0.10%	NA	NA	NA	Only in NCBI
Azithromycin / erythromycin / spiramycin / telithromycin	15	*mph(A*)	1.5	NA	NA	NA	Only in NCBI
Azithromycin / erythromycin / streptogramin	26	*msr(E*)	2.59%	NA	NA	NA	Only in NCBI
Bleomycin	52	*ble, bleO*	5.19%	NA	NA	NA	Only in NCBI
Carbapenem	171	*blaIMP, blaIMP-27, blaKPC-2, blaKPC-3, blaNDM-1, blaNDM-4, blaNDM-5, blaNDM-50, blaNDM-6, blaNDM-7, blaOXA, blaOXA-23, blaOXA-48, blaVIM-1, blaVIM-78*	17.07%	NA	NA	NA	Only in NCBI
Florfenicol	61	*floR, floR2*	6.09%	NA	NA	NA	Only in NCBI
Fosfomycin	14	*fosA, fosA3*	1.40%	NA	NA	NA	Only in NCBI
Gentamicin	54	*aac(3)-IId, aac(3)-IIe, aac(3)-VIa, aac(6′)-Ib, aac(6′)-Ib4, armA*	5.39%	NA	NA	NA	Only in NCBI
Gentamicin / kanamycin / tobramycin	72	*ant(2'')-Ia*	7.19%	NA	NA	NA	Only in NCBI
Hygromycin	13	*aph(4)-Ia*	1.30%	NA	NA	NA	Only in NCBI
Kasugamycin	1	*aac(2')-IIa*	0.10%	NA	NA	NA	Only in NCBI
Lincosamide	42	*lnu(F), lnu(G*)	4.19%	NA	NA	NA	Only in NCBI
Spectinomycin / streptomycin	1	*aadA27*	0.10%	NA	NA	NA	Only in NCBI

^*a*^ NA, not applicable; gene not detected in clinical isolates.

Chloramphenicol resistance was detected in 952 (95%) of the genomes, with 748 harboring the *catA* gene. Additionally, 69 genomes harbored both *catA* and *catA1,* 43 harbored *catA* and *floR,* and the presence of all three genes was seen in 15 genomes (Fig. 4b; Table S6). These findings highlight the prevalence of phenicol resistance in *P. mirabilis* genomes despite the limited use of chloramphenicol in modern clinical settings.

Resistance to aminoglycosides, a critical class of antibiotics for treating severe gram-negative infections, was predicted in 716 (71%) of the genomes. Notably, we observed streptomycin resistance genes in 352 genomes, suggesting the persistence of resistance despite limited clinical use (Table 3). The presence of multiple aminoglycoside resistance determinants indicates that *P. mirabilis* maintains a robust arsenal against this class of antimicrobials, including amikacin, gentamicin, kanamycin, streptomycin, and tobramycin.

Resistance to trimethoprim (TMP), mediated by *dfrA* alleles, was detected in 290 (29%) genomes. The most prevalent allele was *dfrA1* (*n* = 248), followed by *dfrA17* (*n* = 44), and *dfrA32* (*n* = 17); 235 (81.02%) genomes had a single *dfrA* gene, while 50 (17.2%) carried 2, and 5 (1.72%) carried 3 *dfrA* genes (Fig. 4c). The diversity of *dfrA* genes highlights the adaptive potential of *P. mirabilis* to acquire and maintain resistance determinants. Resistance to sulfonamides (SMX) was similarly predicted in 286 (28%) genomes, with *sul1* detected in 180, *sul2* in 187, and 81 genomes harboring both *sul1* and *sul2* genes (Fig. 4d; Table S6). Importantly, 223 of the 363 genomes encoding resistance genes for either TMP or SMX had resistance determinants for both (Fig. 4e). Since TMP and SMX are often used in combination, the co-occurrence of these genes would likely complicate the efficacy of combined treatment. Additionally, we detected 185 genomes (18%) with extended spectrum β-lactamase (ESBL)-associated genes. Of these, 148 harbored the *blaTEM-1* gene, expected to confer resistance to ampicillin (Table 3).

Finally, the number of resistance subclasses detected per *P. mirabilis* genome ranged from 1 to 24, with an average of 4.61 AMR subclasses per isolate. A total of 71 genomes (7%) exhibited resistance to only a single antibiotic subclass, most commonly tetracycline. In contrast, 931 (93%) had resistance to two or more subclasses, and a substantial subset of 246 genomes (25%) harbored more than six antibiotic subclasses, indicating a high prevalence of multidrug resistance in this sample set; 176 genomes (18%) had at least one resistance determinant from all five of the above resistance gene groups, while 56 (6%) had combinations of tetracycline, chloramphenicol, and β-lactam resistance genes. Additionally, phylogenetic analyses (Fig. 1a) determined that certain clusters consistently encoded resistance genes to nearly all antibiotic classes shown, such as genomes belonging to ST135, while others exhibited a complete absence of resistance genes other than tetracycline, independent of study source.

### Antibiotic resistance subclasses are lineage-associated but not strictly determined by ST

To evaluate the relationship between ST and AMR burden in *P. mirabilis*, we quantified the number of predicted AMR subclasses per genome. Logistic regression confirmed that MLST was not a significant driver of high AMR carriage overall (OR 0.0997, 95% CI 0.992–1.002, *P* = 0.304). However, striking lineage-specific enrichment was observed with stratification by resistance burden. Hence, to enable robust comparisons, we focused on a subset of 422 genomes representing the 16 STs with ≥10 genomes each. This accounted for 41% of the data set (Tables S7 and S8). Within this subset, the mean AMR subclass count was 4.4 per genome, with most genomes carrying modest resistomes (80th percentile ~6 subclasses).

Genomes encoding resistances to ≥6 distinct subclasses were enriched in 5 STs (121/142 [85%]): ST135 (37/37 genomes [100%]), ST185 (33/47 [80%]), ST279 (10/12 [83%]), ST97 (25/36 [69%]), and ST145 (16/32 [50%]) (Pearson χ² *P* < 0.001). Among these 142 genomes, 41 (29%) carried resistance genes to ≥12 subclasses, 33 of which (80%) belonged to ST135. In contrast, ST145, ST93, ST33, and ST202 typically carried 2–3 resistance subclasses with some outliers, highlighting interlineage heterogeneity (Table S8). We acknowledge that sampling bias is possible, as many surveillance studies only pick up on hospitalized or severe infections, but our data set also includes general isolate submissions from the CDC surveillance study, and we are most interested in identifying clinically relevant strains.

ST135 emerged as an exceptionally high-risk clone; 95% of ST135 isolates (35/37) harbored ≥16 AMR genes, and 100% carried resistance to ≥6 subclasses. We next examined whether geographic origin confounded this association and created a bias. Within the 422-genome subset, 325 genomes had associated location data (US states/cities), and 167 were from St. Louis. Although a slightly higher proportion of St. Louis isolates exhibited high AMR carriage (≥10 subclasses) compared to other locations (3.4% vs. 0.9%; Pearson χ² *P* = 0.037), isolation source was not an independent predictor in multivariable logistic regression controlling for MLST (OR 1.31, 95% CI 0.88–1.97, *P* = 0.185). The strong enrichment of high-level AMR in ST135 regardless of geographical location points to lineage-intrinsic genetic factors as the primary drivers.

### The distribution of resistance gene burden is consistent across the data set

To contextualize our 26 clinical isolates and HI4320 within the broader *P. mirabilis* population, we compare their AMR gene content against the 1,001 NCBI genomes. In total, resistance genes representing 14 different antibiotic subclasses were identified in the clinical isolates compared to 24 from the NCBI data set. The 26 clinical isolates and reference strain HI4320 (a total of 27) exhibited substantial variation in resistance gene burden, ranging from one gene (minimum, observed in ST765 and ST591 isolates carrying only a tetracycline resistance determinant) to 15 genes (maximum, in ST135 isolate H115-00, H115-29, and H127-00), with an average of 3.8 resistance genes per isolate (Table 3; Table S9).

Despite these differences in data set size and diversity, several resistance traits were highly conserved across both data sets. Tetracycline resistance approached fixation; *tetJ* was present in all isolates predicted to be tetracycline resistant from both data sets, and co-occurrence of *tetJ* with *tetA and tetC* (11%, 3/26 clinical) was also observed. Similarly, chloramphenicol resistance genes demonstrated strong cross-reservoir conservation, with *catA* (type-4) detected in 89% (23/26) of clinical isolates compared to 95% of NCBI genomes, and 26% of clinical isolates (6/23) co-harbored *catA1,* while 11% (3/26) harbored no chloramphenicol resistance genes (Table S9). ST135 strains also exhibited a rare combination of *floR + catB3*.

Fewer of the clinical isolates had resistance genes for cephalosporins or quaternary ammonium in comparison to the NCBI data set (Table 3). However, clinical isolates showed enrichment of resistance genes under UTI treatment pressure, including TMP-SMX (22%, *sul1/sul2*; 37%, *dfr*) and aminoglycosides (streptomycin (37%, most common: *aadA1*), streptothricin (37%, *sat2*), and kanamycin (26%, *aph(3′)-Ia*) (Table 3). In terms of extended β-lactam ecology, clinical isolates predominantly carried *blaTEM-1* (18%), while the NCBI genomes exhibited broader cephalosporin resistance genes (Table 3; Table S6). Collectively, these comparisons reveal a conserved genetic backbone of core resistance determinants (tetracycline and chloramphenicol) across *P. mirabilis* populations, upon which lineage-specific variation is superimposed.

### The mobile genetic element (MGE) landscape of *P. mirabilis* reveals an IS26-mediated major gene cluster in ST135

**Fig 5:**
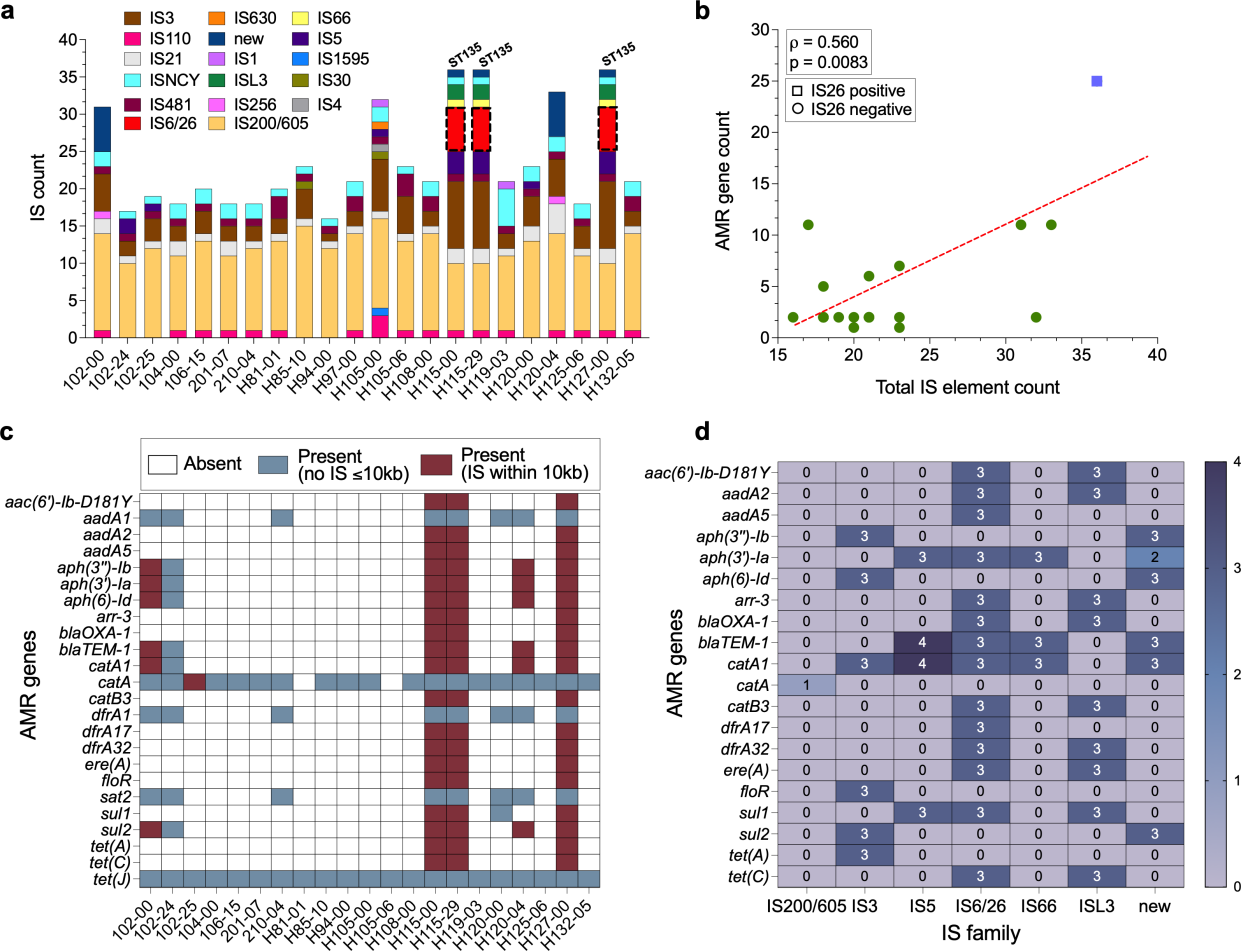
IS element landscape overview of clinical isolates. (**a**) Stacked bar chart of IS family distribution across STs. (**b**) Scatter plot of total IS count vs. AMR gene count with Spearman correlation line (ρ = 0.560, *P* = 0.008). (**c**) Proximity heatmap showing IS26 distance to each AMR gene in all isolates. (**d**) Co-occurrence of IS family-AMR genes in isolates with IS within 10 kb of AMR genes.

To determine if MGEs are associated with AMR gene dissemination in *P. mirabilis*, we characterized 22 complete, polished reference-quality genomes from the clinical cohort. We carried out MGE analysis only using complete, polished genomes for high accuracy. MGE was not done for short-read assembled genomes of the NCBI database, as it is not feasible to obtain an accurate picture from segmented (incomplete) genomes. Among the 22 isolates, a median of 22 IS elements per genome was detected (mean = 23.68; range 16–36) that were distributed across 17 IS families (Fig. 5a; Table S7). The most abundant family was IS200/IS605 (48.5%), followed by IS3 (16.31%) and ISNCY (7.2%). The three isolates with the highest total IS count (36 each) were ST135 (H115-00, H115-29, and H127-00).

The high AMR gene count observed in the three ST135 isolates is positively correlated with IS element count (Fig. 5b). A large AMR gene cluster comprising 14 genes (*aadA2–ere(A)–dfrA32–sul1–arr-3–catB3–blaOXA-1–aac(6′)-Ib-D181Y–tet(C)–sul2–aph(3′′)-Ib–aph(6)-Id–tet(A)–floR*) was associated with flanking IS6 (also known as IS26) (Fig. 5c and d). This cluster spanned ~72.6 kb of contiguous AMR gene content within an IS-bounded region of ~180 kb. IS26 elements (three copies, Fig. 5d) were positioned within and flanking this cluster, with the closest IS26–AMR gene distances of 88–956 bp for six clinically important resistance genes, including *aac(6′)-Ib-D181Y* (92 bp), *aph(3′)-Ia* (88 bp), and *sul1* (112 bp). This organization closely matches the *PmGRI1* genomic resistance island previously characterized in *P. mirabilis* Cluster-1 strains (64). In contrast, non-ST135 isolates with moderate to low AMR burdens (e.g., ST185 with 11 genes, ST145 with 5-11 genes) lacked IS26 entirely (Fig. 5a). IS elements flanking AMR genes in these isolates belonged primarily to the unclassified “new” family, IS3, and IS5 (Fig. 5d).

A conserved Tn7-associated gene cassette (*aadA1-sat2-dfrA1*) was identified in 9/22 isolates (40.9%), three of which were ST135 (Table S10). This cassette displayed an identical 1,938 bp span and gene order in all positive isolates regardless of phylogenetic lineage, confirming the clonal stability of this segment. Interestingly, the three ST135 isolates also harbored a second AMR gene cluster (*catA1-blaTEM-1-aph(3′)-Ia-dfrA17-aadA5-sul1*) spanning ~14.3 kb with identical gene content and order between genomes.

### The MGE landscape reveals a Type IV secretion-system-type ICE in *P. mirabilis* genomes

**Fig 6:**
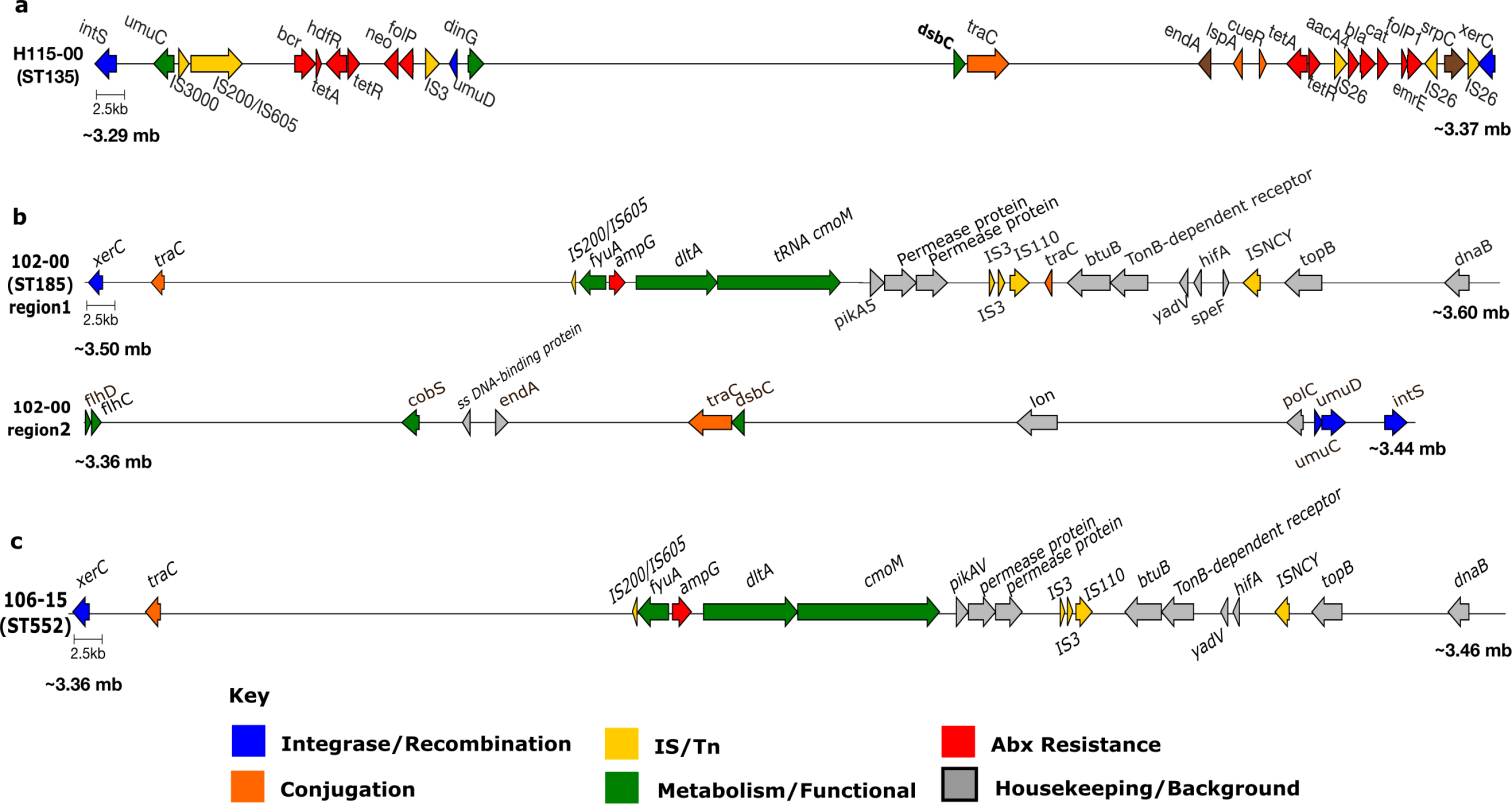
Genetic organization of the T4SS ICEs identified in *P. mirabilis*. (**a**) Genetic structure of the ICE identified in strain H115-00. The element includes the integrase gene intS and regulatory gene umuC, followed by insertion sequence elements (IS3000, IS8 family) and multiple accessory genes. A cluster of antibiotic resistance genes is present, including genes such as bcr*,* tetR, tetB*,* dfrA*,* aac*,* aph, and erm family members, interspersed with transposition- and recombination-related genes (tnpA*,* xerC). (**b**) Two T4SS-ICEs were detected in strain 102-00. Genes are shown as arrows indicating the direction of transcription. The element contains recombination genes recC and uvrC, followed by multiple accessory and housekeeping genes. Conjugation-associated genes, including tra components, are present toward the right side of the element, together with additional metabolic genes. (**c**) T4SS-ICE was detected in strain 106-00. This is a representative for all the non-ST135 and ST145 in our data set. The ICE is similar to the first ICE observed in ST145 in 6b.

Genome mining using ICEberg 3.0 (62) identified 1–2 Type IV secretion system (T4SS)-type ICEs in the 22 complete, polished chromosomes of *P. mirabilis*. The GC content of these elements ranged from 44.86% to 46.19%; they integrated near the tRNA-Phe gene, flanked by identical 52-bp direct repeats (attL/attR), and carried a mobilization protein family H (MOBH) relaxase and a type G mating pair formation system. ST135 isolates (H115-00, H115-29, and H127-00) carried one identical ICE harboring 13 AMR genes along with a copy of the IS26-flanked cluster of 5 genes (*aacA4-blaTEM-catA1-emrE-folP1*) (Fig. 6a). In contrast, ST185 isolates (102-00, H120-04) had two regions with T4SS-type ICEs. Annotation of the ICE identified a range of virulence, housekeeping, and metabolic genes (e.g., permease, PikA5) (Fig. 6b). The other isolates carried only one ICE, harboring similar virulence-associated genes as the region 1 ICE in ST185 (Fig. 6c).

### Prophages and plasmids have a minimal contribution to AMR gene carriage in *P. mirabilis*

PHASTER analysis of the 22 clinical isolate genomes identified 189 prophage regions (mean: 8.2/genome), including 64 intact (33.8%), 20 questionable (10.5%), and 104 incomplete (55%) (Table S10). Coordinate-overlap analysis revealed 14 AMR gene instances within prophage regions across 3/11 isolates (27%), none of which were ST135. The AMR genes in these prophage regions included 10 genes spanning six resistance classes (aminoglycosides, β-lactams, chloramphenicol, sulfonamides, trimethoprim, and disinfectants).

PlasmidFinder detected replicons in only 2/26 isolates (6.7%): IncN4 (conjugative) plus Col3M and ColE10 in H94-00 (ST696), and a single ColE10 in H81-01 (ST765) (Table S10). The only confirmed plasmid-borne AMR gene across all 30 isolates was *qnrD1* (quinolone resistance) on the Col3M plasmid in H94-00 (ST696). This indicates that the overwhelming majority of AMR genes in this *P. mirabilis* collection are chromosomally encoded.

### Hierarchical MGE overlaps reveal a "Russian doll" architecture driving multidrug resistance in ST135

**Fig 7:**
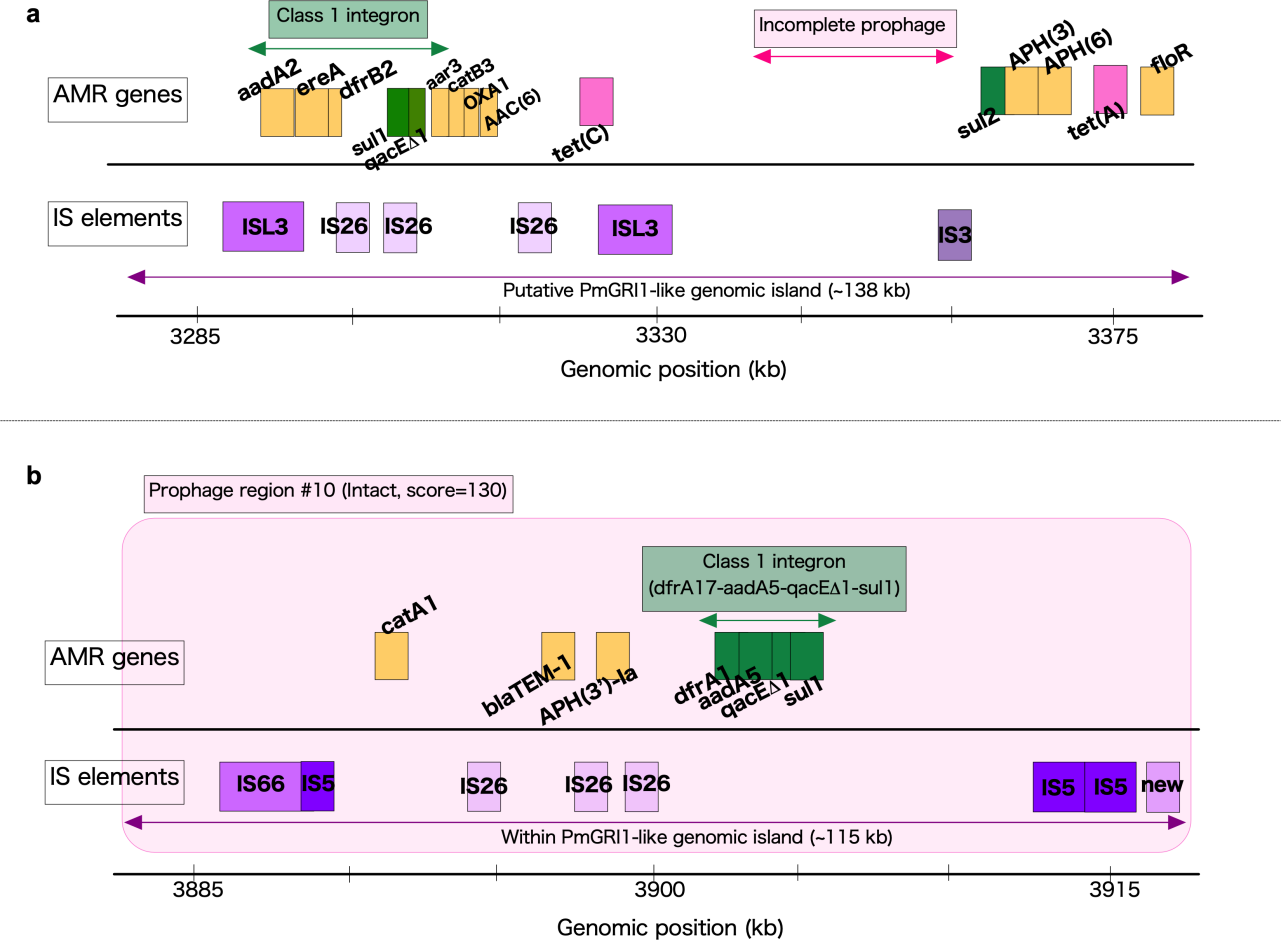
Comprehensive map of the *P. mirabilis* Genomic Resistance Island 1 (PmGRI1)-like region in H115-00 (ST135). (**a**) Putative PmGRI1 region 1 (3.29–3.37 Mb) and (**b**) putative PmGRI1 region 2 (3.885–4.0 Mb). PmGRI1 is a chromosomally integrated, modular resistance island identified by tyrosine-family site-specific recombinase/integrase (~394 aa), insertion at the 3′ end of tRNA-Sec, and 20 bp direct repeats flanking the element.

Systematic analysis of MGE overlaps within the 22 clinical isolates revealed a nested hierarchical architecture most pronounced in ST135 isolates. Class 1 integrons with their 3′-CS (*qacEΔ1-sul1*) are embedded near IS26 sites. Each integron carries distinct aminoglycoside and trimethoprim resistance cassettes (*aadA, dfrA*). These modules are themselves clustered within *PmGRI1***-**like genomic islands containing IS elements. In ST135 isolates, IS26 serves as the structural organizer of *PmGRI1*: Region 1 contains 6 IS elements (3 × IS26, 2 × ISL3, and 1 × IS3) interspersed among 16 AMR genes (Fig. 7a); Region 2 contains 8 IS elements (3 × IS26, 3 × IS5, IS66, and IS200/IS605) among 10 AMR genes (Fig. 7b). IS26 may act as a boundary element for sub-modules within *PmGRI1*, enabling modular rearrangement and stepwise accumulation of resistance. In the same arrangement, Prophage region 10 (35.1 kb, intact, score 130, with *att* sites) simultaneously overlaps region 2. This represents a four-way overlap of all element types within a single 35 kb region, nested within *PmGRI1*.

### Experimental AMR phenotyping of *P. mirabilis* clinical isolates reveals variable genotype-phenotype concordance

**Fig 8:**
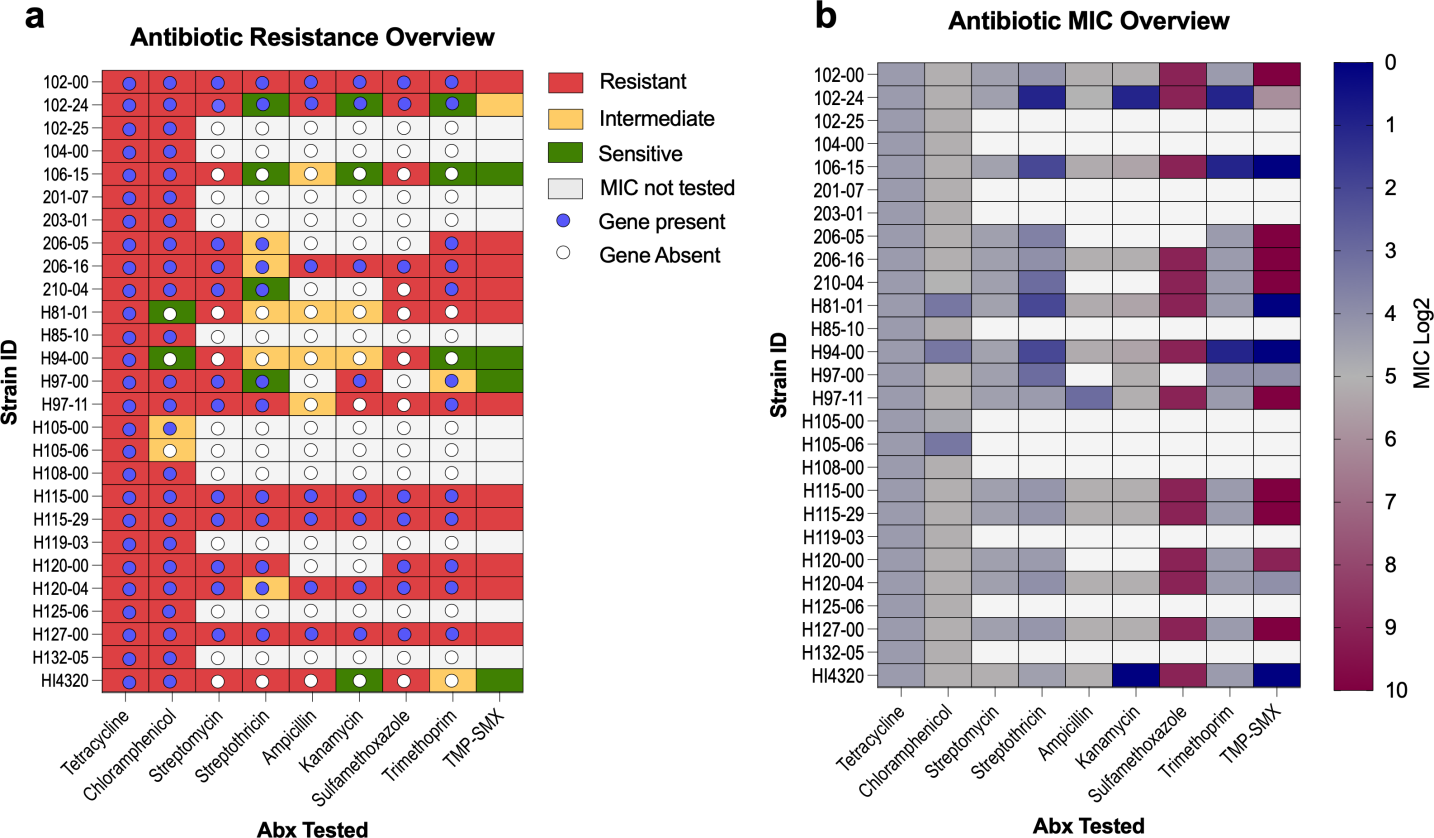
Overview of antibiotic resistance and MIC. (**a**) Overview of antibiotic resistance phenotype found in the clinical CAUTI isolates. Color coding denotes resistance status: red = resistant, yellow = intermediate resistance, and green = susceptible strains. The presence or absence of resistance-associated genes is indicated by filled and empty circles, respectively. (**b**) Heatmap representing the MIC for each antibiotic tested across clinical *P. mirabilis* CAUTI isolates. Color intensity corresponds to MIC values, indicating variation in resistance levels.

**Fig 9:**
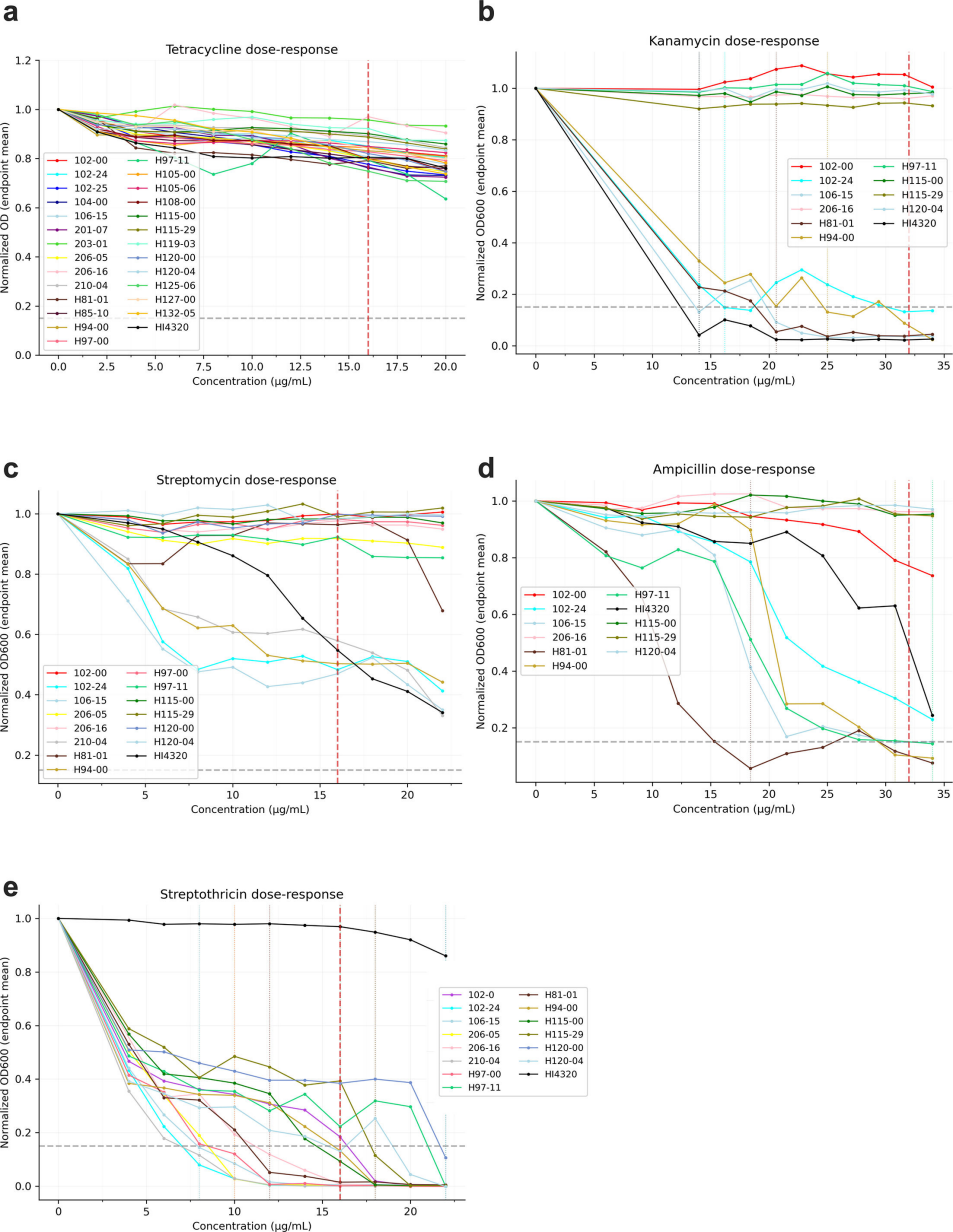
Dose-response curves and MIC. Mean normalized OD600 values at experimental endpoint (20 h) are plotted against antibiotic concentrations in µg/mL. OD is normalized to a no-drug control set to 1.0 (maximum growth). The red vertical line indicates the resistance breakpoint (CLSI) for *Enterobacterales*. The MIC was determined as the lowest antibiotic concentration for which growth was restricted below a threshold of a normalized OD of 0.15 (gray horizontal dashed line). (**a**) Tetracycline (MIC range tested: 2–20 µg/mL), (**b**) Kanamycin (MIC range tested: 6–34 µg/mL), (**c**) Streptomycin (MIC range tested: 4–22 µg/mL), (**d**) Ampicillin (MIC range tested: 6–34 µg/mL), and (**e**) Streptothricin (MIC range tested: 4–22 µg/mL).

AMRFinderPlus reports gene presence/absence and point mutation, but does not infer phenotypic resistance due to factors including gene expression and regulation. Thus, many of the resistance genes detected by established computational databases may not be relevant for clinical management or antimicrobial surveillance. To determine concordance between *in silico* AMR predication and phenotypic resistance in *P. mirabilis*, we determined the AMR of all 27 *P. mirabilis* clinical isolates by standardized antimicrobial susceptibility tests (AST). We tested the growth kinetics of strains with specific resistance genes at 15-min intervals over a 20-h time course to evaluate the minimum inhibitory concentrations as well as more nuanced dose-dependent effects on the growth rates and doubling times of the *P. mirabilis* strains (Fig. 8 and 9). Antibiotic concentrations were selected based on the Clinical and Laboratory Standards Institute (CLSI) guidelines for *Enterobacterales* and were designed to span the thresholds for sensitive (lowest) to resistant (highest). The genotypic and phenotypic resistance profiles are summarized in Tables S9 and S11.

#### Tetracycline, kanamycin, streptomycin, streptothricin, and ampicillin AMR gene carriage predicts phenotypic resistance

For tetracycline, 100% of clinical isolates (26/26) exhibited resistance with MIC values > 20 µg/mL, which is consistent with the presence of *tetJ*. Growth kinetics demonstrated uniformly robust growth across all concentrations (Fig. 9a). Thus, a perfect genotype-phenotype concordance was observed. Carriage of any additional MGE-acquired *tet* genes (*tetA* or *tetC*) is phenotypically redundant.

Kanamycin resistance genes (*aac(6′)-Ib-cr5, aph(3′)-Ia*) were detected in 7/26 clinical isolates, and genotype-phenotype concordance was observed. All seven isolates encoding the resistance genes had MIC > 34 µg/mL, while 106-15, H81-01, H94-04, and HI4320 were predicted to lack all kanamycin resistance determinants and were susceptible, but some exhibited MICs ranging from 14 to 20.5 µg/mL (Fig. 9b).

Different combinations of streptomycin resistance genes (*aadA1, aph(6)-Id, aph(3′′)-lb, aadA5, aadA2*) were observed in 11/26 clinical isolates (Fig. 8a; Table S10), and all provided resistance to streptomycin when tested in our AST assay (Fig. 9c). Unexpectedly, resistance was also observed in 102-24, 106-15, H81-01, H94-00, and reference strain HI4320, which lacks canonical streptomycin resistance genes. However, these strains all exhibited a dose-dependent decrease in growth when incubated with streptomycin. It is also notable that clinical isolate 210-04 (one predicted gene, *aadA1*) exhibited a similar dose-dependent decrease in growth, although other strains (206-05, H97-00, and H97-11) with a singular *aadA1* gene with 100% amino acid sequence similarity to that of 210-04 did not show dose dependency. It is possible that regulation of *aadA1* differs in 210-04, leading to a less efficient or inducible mechanism of resistance compared to the other isolates. The presence of multiple, stacked aminoglycoside resistance genes on MGEs like *PmGRI1* in H115-00 (e.g., *aadA1, aph(6)-Id, aph(3′′)-lb*) can confer broader and potentially higher-level resistance compared to strains carrying only a single gene, explaining quantitative differences in MICs beyond simple gene presence.

An ampicillin resistance gene (*blaTEM*) was identified in 6/26 clinical isolates, and all exhibited MICs > 34 µg/mL. The resistance phenotype was also observed in HI4320, which lacks any known β-lactamase genes. However, strains that did not encode *blaTEM*, including HI4320, exhibited dose-dependent growth inhibition with increasing concentrations of ampicillin, despite displaying resistance at CLSI breakpoints (Fig. 9d). The dose-dependent growth inhibition observed in some strains with *bla*TEM, and the low-level resistance in strains lacking it, may be due to IS-proximal regulatory effects influencing gene expression or the presence of alternative, non-canonical resistance mechanisms located within genomic islands.

Streptothricin resistance gene *sat2* was observed in 11/26 clinical isolates (Fig. 8a; Table S9); however, the AST assay revealed a mix of intermediate to resistant phenotypes with MICs ranging from eight to > 22 µg/mL (CLSI breakpoints are ≤ 8 for susceptible and ≥ 16 for resistant). Since streptothricin resistance is conferred by *sat1, sat2*, and *sat4* genes, the presence of only one gene may confer only partial resistance, with dose-dependency increasing with gene stacking. The genotypically positive isolates 102-00, 206-16 H97-11, H115-00, H115-29, H120-00, and H120-04 exhibited dose-dependent decreases in growth with increasing streptothricin concentrations. However, other genotypically positive strains 206-05 and 210-04 were sensitive (Fig. 9e). HI4320 exhibited robust growth regardless of streptothricin concentration; however, this strain does not encode any known streptothricin resistance genes (Fig. 9e).

#### Sulfonamide and trimethoprim AMR gene absense does not predict phenotypic susceptiblity

**Fig 10:**
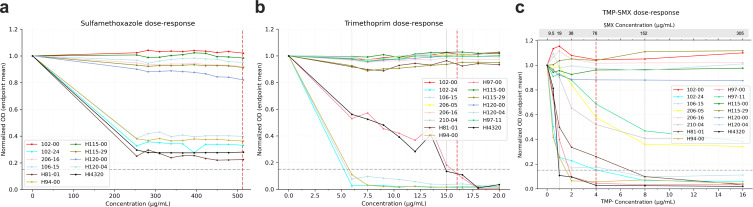
Dose-response curves and MIC. Mean normalized OD600 values at experimental endpoint (20 h) are plotted against antibiotic concentrations in µg/mL. OD is normalized to a no-drug control set to 1.0 (maximum growth). The red vertical line indicates the resistance breakpoint (CLSI) for *Enterobacterales*. The MIC was determined as the lowest antibiotic concentration for which growth was restricted below a threshold of a normalized OD of 0.15 (gray horizontal dashed line). (**a**) Sulfamethoxazole (MIC range tested: 250–512 µg/mL), (**b**) Trimethoprim (MIC range tested: 4–22 µg/mL), (**c**) TMP-SMX (MIC range tested: 0.5/9.5–16/305 µg/mL).

Sulfonamide resistance genes (*sul1* and *sul2*) were both observed in the 26 clinical isolates, with *sul1* present in three isolates (H115-0, H115-05, and H120-00), *sul2* in five isolates (102-00, 206-16, H120-04, H115-00, and H115-05), and both genes co-occurring in three isolates (H115-00, H115-05, and H127-00) (Table S9). AST with sulfamethoxazole revealed high-level resistance (MIC > 514 µg/mL) in all isolates, irrespective of *sul* gene carriage (single or double), indicating that the presence of an additional *sul* gene did not further elevate resistance. Unexpectedly, even strains that did not encode *sul* (102-24, 106-15, 210-04, H81-01, H94-00, H97-11, and HI4320) had MICs > 514 µg/mL, but partial growth inhibition was clearly present even at the lowest tested concentration (250 µg/mL, Fig. 10a). The uniform MIC > 514 µg/mL across all strains implies clinical resistance per CLSI guidelines, as it far exceeds the susceptible breakpoint (typically ≤ 38 µg/mL). However, the partial growth inhibition of strains that do not encode *sul* highlights the complexity of interpreting MICs near the resistance threshold. These data suggest that while canonical *sul*-mediated resistance mechanisms were absent in these isolates, IS elements located near AMR genes or regulatory regions may contribute to the observed resistance (above threshold) phenotype.

Trimethoprim resistance genes were also detected in 10/26 clinical isolates, with MICs of > 15 µg/mL (exceeding clinical breakpoints) regardless of which resistance gene (*dfrA1*, *dfrA17*, or *dfrA32*) was present (Fig. 10b). However, one isolate (H97-00) harboring *dfrA1* exhibited dose-dependent growth inhibition, contrasting with other strains harboring *dfrA1* with 100% amino acid similarity that showed no dose dependency. This implies strain-specific phenotypic variability (e.g., differential gene expression, compensatory mutations) despite shared genetic determinants. The *dfrA*-negative reference strain H81-01 also displayed full resistance, while strain HI4320, which did not carry the *dfrA* gene or gene mutation, displayed intermediate resistance and mirrored the dose-dependent growth inhibition of strain H97-00. These observations support the existence of non-*dfrA* resistance mechanisms in HI4320 and H81-01, such as altered folate metabolism or reduced drug uptake. The discordance between genetic markers and phenotypic outcomes also underscores a key challenge: genetic testing alone cannot always predict antibiotic response. However, *dfr* negative strains 102-24, 106-15, and H94-00 were sensitive to trimethoprim, in concordance with the genotype.

Since the genotype-negative strains were all phenotypically resistant to sulfamethoxazole (SMX), albeit with reduced growth rate, we further tested the impact of combinatory treatment (trimethoprim-sulfamethoxazole, TMP-SMX) on *P. mirabilis* growth. This is an important parameter since, historically, TMP-SMX was the first-line empirical treatment for acute uncomplicated UTIs. In the strains exhibiting 100% genotype-phenotype concordance for both SMX and TMP resistance (i.e., true resistant genotypes correlated with resistant phenotypes), all isolates (102-00, 206-16, H115-00, H115-05, H120-00, and H120-04) exhibited complete resistance to the combinatory drug without any dose-dependent decreases in growth (Fig. 10c). In contrast, strains such as 206-05, 210-04, and H97-11 that were genotypically SMX-susceptible, TMP-resistant, but phenotypically resistant to both antibiotics when used alone exhibited a dose-dependent resistance phenotype when treated with TMP-SMX. Additionally, strains that were genotypically predicted to be sensitive to both antibiotics but were phenotypically resistant demonstrated intermediate to resistant phenotypes for combination treatment: H81-01 displayed an MIC of 8/152 µg/mL (resistant), while 102-24 displayed an MIC > 2/38 µg/mL (intermediate). This is clinically concerning, as growth was observed at breakpoint concentrations. Other discordant strains that had lower growth yields under single SMX exposure (106-15, H94-00, H97-00, and HI4320) were found to be sensitive to the TMP-SMX combinations (Fig. 10c).

#### Chloramphenicol AMR gene carriage predicts phenotypic resistance, and gene stacking further increases resistance

**Fig 11:**
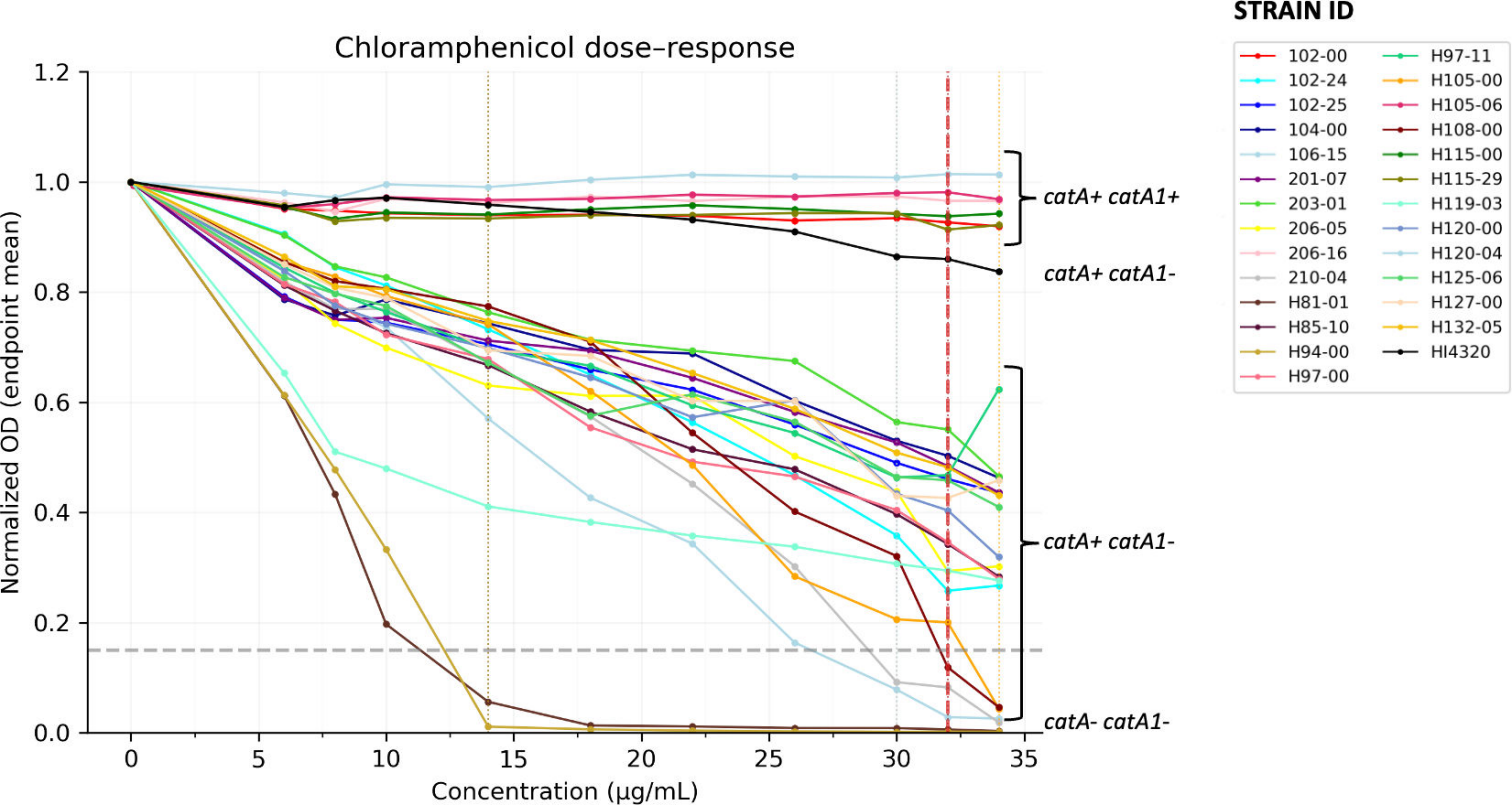
Dose-response curves and MIC of chloramphenicol. Mean normalized OD600 values at experimental endpoint (20 h) are plotted against antibiotic concentrations in µg/mL. OD is normalized to a no-drug control set to 1.0 (maximum growth). The red vertical line indicates the resistance breakpoint (CLSI) for *Enterobacterales*. The MIC was determined as the lowest antibiotic concentration for which growth was restricted below a threshold of a normalized OD of 0.15 (gray horizontal dashed line). Chloramphenicol concentrations tested ranged from 6 to 34 µg/mL.

Chloramphenicol resistance displayed genotype-phenotype concordance, with all isolates possessing a *catA* gene exhibiting resistance and those lacking *cat* genes exhibiting susceptibility (Fig. 11). MIC values ranged from 10 to 14 µg/mL in isolates lacking a *cat* gene but were > 26 µg/mL in isolates with *catA* alone and > 34 µg/mL in isolates harboring both *catA* and *catA1* (Table S9). Interestingly, many isolates with a single *catA* gene (16/17) exhibited dose-dependent growth inhibition (Fig. 11), while isolates possessing both *catA* and *catA1* demonstrated hyper-resistance (hR) with no dose dependency (Fig. 11). The exception was HI4320, which only encodes *catA* but exhibited robust growth at all but the highest three concentrations of chloramphenicol. No strains were predicted to have *catA1* alone; hence, we were not able to determine whether *catA1* provides a greater level of resistance than *catA,* but our data suggest that the presence of two *catA* genes enhances functional resistance.

We next compared the sequences of the *catA* and *catA1* genes in our *P. mirabilis* clinical isolates that harbored both genes, as *catA1* has not previously been reported in *P. mirabilis*. The two genes share 76% amino acid sequence identity (with representative strain 102-00; Fig. S2a), and *catA* from strain 102-00 showed 100% identity to the *catA* gene in reference strain *P. mirabilis* HI4320 (Fig. S2b). The nucleotide and amino acid sequences of *catA1* from our isolates share 100% amino acid identity with *catA1* from *E. coli* and ~98% amino acid identity with *catA1* from *Photobacterium damselae* and *Shigella flexneri* (Fig. S2c).

Although we had a limited sample size, within-lineage discordance was observed in ST145 and can be attributed to differential MGE acquisition. Strain 102-24 carries 11 AMR genes (including IS5-flanked accessory cluster with *aph(3′′)-Ib, aph(3′)-Ia*, *aph(6)-Id, blaTEM-1, catA1,* and *sul2*), while 210-04 carries only five genes. The six additional genes in 102-24 are MGE-acquired (flanked by IS5 elements), conferring predicted resistance to 3+ additional drug classes. This means two isolates of the same sequence type would have different AST profiles, a difference that is possibly explained by differential MGE acquisition.

## DISCUSSION

CAUTIs caused by *P. mirabilis* remain clinically challenging due to crystalline biofilm formation, recurrent infections, and persistence despite antibiotic treatment and catheter changes (15). This comprehensive genomic analysis of *P. mirabilis* from human urinary sources expands our understanding of *P. mirabilis* genetic diversity, AMR profiles, and the accessory genome. We analyzed 1,027 publicly available and clinical isolates and determined that urinary *P. mirabilis* isolates possess an open pangenome with more than 41,121 gene families and a core genome of 1,937 genes. Open pangenomes are characteristics of sympatric species with a high capacity of horizontal gene transfer (HGT) mediated by MGEs, highlighting genomic plasticity. This may allow *P. mirabilis* to rapidly adapt to the urinary tract under antibiotic exposure, host immune responses, and competition in the polymicrobial catheterized environment (65–67). This dynamic gain, loss, and exchange of MGEs with accessory genomes comprising ~90% (37,918 genes) emphasizes the importance of flexible genomic elements in driving diversity in the urinary tract.

The presence of 213 defined STs and ~118 previously undefined genomes and a star-like phylogeny further supports ongoing diversification. Almost half of the STs were represented by single isolates (46%), showcasing heterogeneity. However, most genomes were distributed across a small subset of STs (ST96, ST185, ST99, ST135, and ST97), resulting in a skewed distribution. While MLST remains a widely used typing method due to its reproducibility and interpretability (51, 68), we want to highlight its limitations for epidemiological investigations (68, 69). The substantial proportion of unidentified STs and limited sample size constrain ST-specific characterization in the entire 1,027 genomes but highlights the need for continuously updated typing schemes.

The AMR landscape was complex and dynamic, with variable genotype-phenotype concordance. Overall, 93% of the isolates were resistant to ≥ 2 antibiotic subclasses and 25% resistant to > 6 subclasses, posing a challenge for effective treatment of *P. mirabilis* UTIs. Although MLST was not a significant predictor of high AMR carriage in the full data set of 1,001 genomes, ST135 emerged as a high-risk clone in the subset of 422/1,001 with > 10 genomes; 95% of ST135 genomes had ≥ 16 resistance genes, and 100% had resistance to > 6 antimicrobial subclasses (Fig. 1a). This near-uniform high-level resistance within ST135 is attributed to the IS26-mediated MGEs that are rarely observed in other lineages. IS6/IS26-encoding genomes carry 3.2× more AMR genes (20.8 vs. 6.5) and 2.8× more drug subclasses (13.9 vs. 4.9), with a significant dose-response relationship as IS6 copy number increases. MLST ST135 had the highest IS diversity in our data set (37/422 genomes + 3 clinical isolates), the highest IS6/IS26 prevalence, and AMR gene burden with 100% MDR (>3 antibiotic subclasses).

Geographic confounding was excluded by our analysis; although ST135 was predominantly isolated from St. Louis, isolation source alone was not an independent driver of high AMR carriage (*P* = 0.051), pointing to lineage-intrinsic factors rather than regional antibiotic selection pressures. The absence of geographical associations, together with the hierarchical MGE architecture we identified (Fig. 7), implicates lineage-specific MGE carriage as the mechanistic basis for extreme resistance phenotypes. This is known for other species as well—*S*. *aureus* USA300 (ST8/5) carries the mecA cassette and is seen globally (70–72). The overlapping MGE architecture (H115-00; Fig. 7) reveals a multi-level system for AMR propagation. Class 1 integrons capture individual resistance cassettes, IS26 organizes these into modular translocatable units, *PmGRI1* provides stable chromosomal integration, and prophages enable the horizontal transfer of complete AMR modules. This nested hierarchy has clinical implications because redundant dissemination pathways can ensure robust AMR spread. Clustering of multiple resistance genes within shared elements means selection for any single antibiotic co-selects the entire MDR module, and chromosomal integration ensures stable inheritance, while IS26 and phage mechanisms retain mobility. The exclusive association of this architecture with ST135, which carries significantly more AMR genes than IS26-negative lineages (mean 25.0 vs. 4.1, *P* = 0.0019), implicates these overlapping MGEs as key drivers of this lineage’s epidemiological success.

A recent study by Zhang et al. (64) identified the same high-risk lineage, which they assigned Cluster-1 genomes comprising 233 globally distributed isolates (64). Our analysis confirmed that Cluster-1, which mostly carries ST135 (159/233), had significantly elevated AMR gene burden. The high AMR gene counts across isolates from different locations and hosts that were reported by *Zhang* et al*.* further supports ST135 as a lineage of major clinical concern (64). The T4SS ICE and IS26-mediated AMR gene clusters help us to stack AMR genes within *PmGRI1*, enabling vertical transmission and potentially facilitating further resistance acquisition. Such lineage-specific AMR enrichment has critical implications for infection control and genomic surveillance, as clonal expansion of ST135 could rapidly disseminate multidrug resistance across healthcare networks, similar to methicillin-resistant *S. aureus* (MRSA) and vancomycin-resistant *E. faecalis* (VRE) (70, 72–78). Prospective surveillance should therefore prioritize tracking this high-risk lineage, and future functional studies should dissect the precise MGEs responsible for its resistance burden and why it is limited to this specific lineage. In contrast, the broader distribution of common resistance subclasses across multiple STs reflects ongoing horizontal gene transfer. These findings highlight the critical need for genomic surveillance targeting high-risk lineages, as clonal expansion of ST135 could rapidly disseminate multidrug resistance across healthcare networks.

The overwhelming prevalence of tetracycline resistance genes (98.9%), particularly *tetJ*, aligns with the intrinsic resistance of *P. mirabilis*. The dominance of *tetJ* and the frequent carriage of only a single *tet* gene (94.3%) suggest that this determinant is often sufficient for the observed resistance phenotype. Tetracycline resistance also exemplifies how MGE-acquired genes can be phenotypically redundant: *tet*(J) alone confers complete resistance (MIC > 20 µg/mL); however, some genomes, such as the ST135 isolates, carry three genes (*tet*(J), *tet*(A), *tet*(C)) acquired on the PmGRI1 genomic island via IS26. Thus, the additional MGE-borne genes inflate genotypic counts without meaningfully expanding the phenotype.

Chloramphenicol resistance genes (*catA*, 95%) were prevalent despite limited clinical use, possibly reflecting co-selection with other antibiotics or acquisition due to extensive use in agricultural and environmental settings (79, 80). The co-occurrence of *catA* and *catA1* was detected in 19.2% of *cat*-positive isolates, with rare *floR* and *catB3* gene stacking. Strains with only a single *cat* gene exhibited dose-dependent growth inhibition, while additional *catA1* resulted in a hyper-resistant phenotype (no growth inhibition at all at any tested concentrations), enhancing the resistance phenotype. This observation suggests that the combined expression of multiple acetyltransferases, driven by IS26 elements positioned within 10 kb, quantitatively enhances resistance. Detection of *catA1* in *P. mirabilis* is a significant finding, as this gene has not been well reported and characterized in *P. mirabilis* but matches closely to related bacterial species (81, 82).

Additionally, we observed resistance to aminoglycosides (71%) with streptomycin resistance often co-occurring with other determinants of carbapenems, cephalosporins, fluoroquinolones, and trimethoprim-sulfonamides (Fig. 1). This persistence despite reduction in clinical use may reflect MGE-mediated clonal spread of resistant lineages. Trimethoprim (TMP, *dfr*, 29%) and sulfonamide (SMX, *sul*, 28%) resistance determinants also co-occurred in 22% of genomes, threatening the efficacy of this combination therapy for *P. mirabilis* for UTI treatment. While ESBL-associated genes were less common (18.5% and primarily *blaTEM-1*), their presence is concerning for broader β-lactam resistance development. Resistance for quinolones (*qnr aac(6′)-Ib-cr, gyrA/parC* mutations) and the chloramphenicol/florfenicol (*cat, floR*) combination were rare in both data sets (~7%) but noteworthy as potential molecular fingerprints of human-driven selection linked to agriculture use and MGE gene transfer from *P. mirabilis* lineages found in animal sources (35, 64, 83–86).

Our comprehensive AMR analysis shows a complex genotype-to-phenotype landscape with both expected concordance and clinically critical discordance. Concordance for antibiotics like tetracycline was expected, and kanamycin resistance was reliably predicted by the presence of an *aph(3′)-la* gene, validating the robustness of our genomic approach for identifying known resistance determinants (75, 87, 88). However, discordances provide compelling insights into AMR dissemination. For example, resistance to streptomycin and streptothricin could be due to novel acetyltransferases. Similarly, the ampicillin resistance in strains 102-24, 106-15, H94-00, H97-11, and HI4320 that lack a detectable β-lactamase gene may reflect permeability or efflux mechanisms, which is a hallmark of concentration-dependent antibiotics (89, 90), where higher concentrations or adjuvants can eventually overcome cellular defenses.

A critical finding was the discordance between genotypic SMX susceptibility and TMP-SMX phenotypic response (Fig. 8). Strains that lacked the canonical *sul* genes yet exhibited variable resistance phenotypes could possibly be explained by MGE-related mechanisms: (i) IS elements positioned ≤ 100 bp from AMR genes in seven isolates may drive upregulation via outward-facing promoters, and (ii) ST135 carries three *dfr* genes (*dfrA1*, *dfrA17*, and *dfrA32*) accumulated via IS26 on *PmGRI1*, while some isolates carry only *dfrA1* via non-IS26 Tn7 transposon. Thus, strains like 206-05 and 210-04 (MIC > 16/305 µg/mL) likely possess MGE-driven mechanisms robust enough to contribute to resistance, albeit less efficiently than truly resistant strains, while the sensitivity of strains like HI4320 has mechanisms still overcome by trimethoprim. Such prediction errors can be dangerous for treatment outcomes.

The genotype-phenotype discordance also highlights a significant limitation of current genomic prediction models: their reliance on curated databases of known resistance genes and mutations. Our study confirms that WGS-based prediction is highly reliable for well-characterized resistance determinants in *P. mirabilis* but can fail to predict phenotypes that are likely governed by cryptic mutations, efflux, and MGEs that impact gene expression, copy number, and functional redundancy. Not much has been reported on the mechanism of resistance involving efflux systems and membrane permeability (10, 83, 91). Efflux systems such as *AcrAB-TolC* reported in many *P. mirabilis* strains can be overwhelmed by high drug concentrations, rather than the efficient enzymatic hydrolysis provided by a beta-lactamase and is linked to very high multidrug-resistance rates (92). Genotype-based predictions are also prone to sequencing, assembly, and annotation errors, ideally needing long-read or reference-based sequencing to create hybrid assemblies. However, this can become impractical and costly for diagnostic laboratories.

Several limitations should be considered when interpreting our findings. First, the publicly available genomes were only derived from human hosts in the US, potentially biasing global population inferences. Second, our MGE analysis was restricted to the 22 clinical hybrid-assembled genomes to ensure accuracy, limiting the sample size for these comparisons, but short reads are not an accurate predictor for deep MGE analysis. Third, while we identified compelling associations between MGE architecture in ST135 and AMR burden, it was done only in the sample set of 26 clinical isolate genomes and HI4320 with short-read assembly. Fourth, functional validation of IS26-mediated gene stacking and prophage transduction is necessary to confirm the mechanistic roles proposed here. Finally, our genotype-phenotype discordance analysis, although comprehensive, was necessarily limited by the number of clinical isolates with paired AST data.

In conclusion, while WGS has the potential to be used widely in a clinical context for AMR predictions, our findings reinforce that assessing gene presence/absence does not replace phenotypic AST of *P. mirabilis* isolates for accurate clinical decision-making and surveillance. Accurate resistance prediction requires a larger sampling size, database curation, resistance gene detection, and comprehensive characterization of the MGE landscape in *P. mirabilis*. These findings highlight the need for genomic surveillance targeting both high-risk lineages and the mobile elements that enable resistance gene flux across phylogenetic boundaries.

## Data Availability

Sequencing data have been deposited in NCBI BioProject PRJNA1367153: *Proteus mirabilis* genome sequences from human urine sources in the United States. All other data are available within the article and its supplemental material. All basic commands and databases used are available in the supplementary appendix.

## SUPPLEMENTAL MATERIAL

Data S1 aac.01768-25-s0001.pdf Bioinformatics tools, databases, and commands used in this manuscript.

Supplemental figures aac.01768-25-s0002.pdf Fig. S1 and S2.

Supplemental tables aac.01768-25-s0003.xlsx Tables S1 to S11.

ASM does not own the copyrights to Supplemental Material that may be linked to, or accessed through, an article. The authors have granted ASM a non-exclusive, world-wide license to publish the Supplemental Material files. Please contact the corresponding author directly for reuse.
