# Supplementary material for: Pangenome analysis of Proteus mirabilis reveals lineage-specific antimicrobial resistance profiles and discordant genotype-phenotype correlations
Source: Antimicrob Agents Chemother. 2026 May 29;70(7):e01768-25. doi: 10.1128/aac.01768-25 (PMC13321803; doi:10.1128/aac.01768-25)
Supplement: Supplemental figures — Fig. S1 and S2. [file aac.01768-25-s0002.pdf]

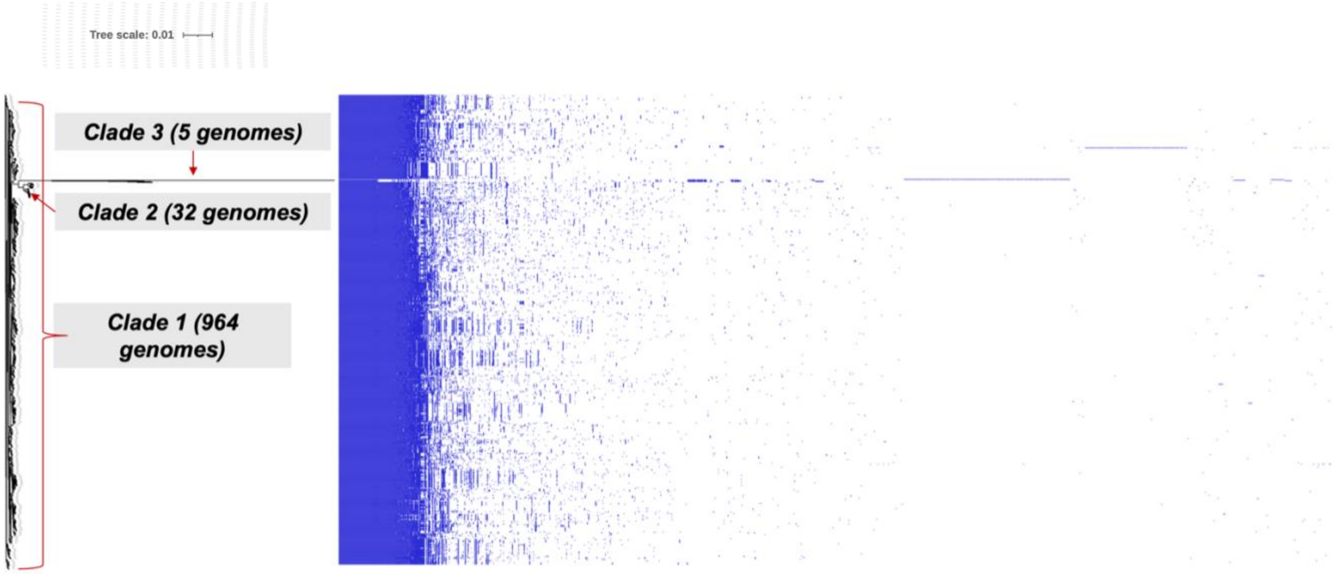

Fig S1. Mosaic pan genome with presence/absence matrix of core-genome phylogenetic tree for 1,001 *P. mirabilis* genomes. Three clades with different branch lengths (*sb1, sb2,* and *sb3*) are represented in the phylogenetic tree, where *sb2* has a clear difference in core genome.

a

```
         1        10        20        30        40        50        60
catA     MDTKRVGYTVVDLSQWGRKEHFEAFQSFAQCTFSQTVQLDITSLLKTVKQNGYKFYPTFI
catA1    MEKKITGYTTVDISQWHRKEHFEAFQSVAQCTYNQTVQLDITAFLKTVKKNKHKFYPAFI

                  70        80        90       100       110       120
catA     YIISLLVNKHAEFRMAMKDGELVIWDSVNPGYTIFHEQTETFSSLWSYYHKDINHFLKTY
catA1    HILARLMNAHPEFRMAMKDGELVIWDSVHPCYTVFHEQTETFSSLWSEYHDDFRQFLHIY

                 130       140       150       160       170       180
catA     SEDIAQYGDDLAYFPKEFIENMFFVSANPWVSFTSFNLNVANINNFFAPVFTIGKYYTQG
catA1    SQDVACYGENLAYFPKGFIENMFFVSANPWVSFTSFDLNVANMDNFFAPVFTMGKYYTQG

                 190       200       210
catA     DKVLMPLAIQVHHAVCDGFHVGRLLNEIQQYCDEGCK..
catA1    DKVLMPLAIQVHHAVCDGFHVGRMLNELQQYCDEWQGGA
```

b

```
               1        10        20        30        40        50        60
catA-pmHI4320  MDTKRVGYTVVDLSQWGRKEHFEAFQSFAQCTFSQTVQLDITSLLKTVKQNGYKFYPTFI
catA-102-0     MDTKRVGYTVVDLSQWGRKEHFEAFQSFAQCTFSQTVQLDITSLLKTVKQNGYKFYPTFI

                        70        80        90       100       110       120
catA-pmHI4320  YIISLLVNKHAEFRMAMKDGELVIWDSVNPGYTIFHEQTETFSSLWSYYHKDINHFLKTY
catA-102-0     YIISLLVNKHAEFRMAMKDGELVIWDSVNPGYTIFHEQTETFSSLWSYYHKDINHFLKTY

                       130       140       150       160       170       180
catA-pmHI4320  SEDIAQYGDDLAYFPKEFIENMFFVSANPWVSFTSFNLNVANINNFFAPVFTIGKYYTQG
catA-102-0     SEDIAQYGDDLAYFPKEFIENMFFVSANPWVSFTSFNLNVANINNFFAPVFTIGKYYTQG

                       190       200       210
catA-pmHI4320  DKVLMPLAIQVHHAVCDGFHVGRLLNEIQQYCDEGCK
catA-102-0     DKVLMPLAIQVHHAVCDGFHVGRLLNEIQQYCDEGCK
```

c

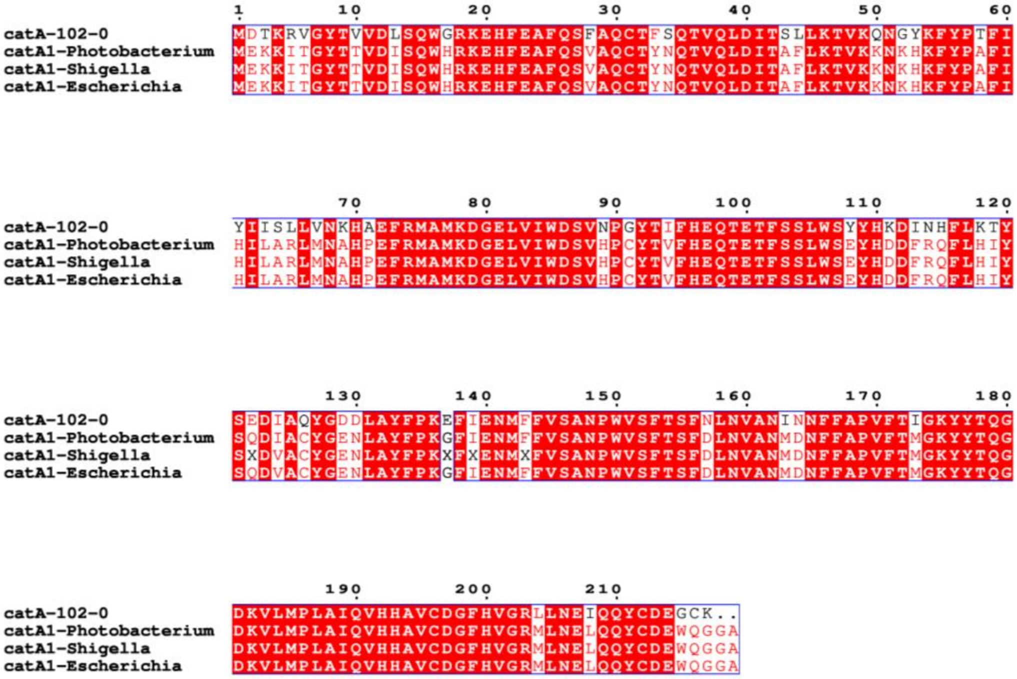

Fig S2. Comparative sequence analysis of *catA* and *catA1* genes in *P. mirabilis* clinical isolates. a. Amino acid equence comparison between *catA* and *catA1* demonstrates 78% amino acid sequence identity. **b.** Sequence comparison between the *catA* gene from strain 102-00 to the *catA* gene of reference strain *P. mirabilis* HI4320 shows 100% amino acid sequence identity. **c**. Sequence comparison between the *catA1* gene in different species of *Enterobacterales*.
